# A Case Study on Measuring AI Assistant Competence in Narrative Interviews

**DOI:** 10.12688/f1000research.151952.1

**Published:** 2024-06-07

**Authors:** Chitat Chan, Yunmeng Zhao

**Affiliations:** 1 Social Work, Hong Kong Baptist University, Hong Kong, Hong Kong

**Keywords:** Artificial Intelligence, Narrative Inquiry, Qualitative Research, WhatsApp Interviews, Conversational AI, Prompt Engineering, Digital Research Methodologies

## Abstract

**Background:**

Researchers are leading the development of AI designed to conduct interviews. These developments imply that AI's role is expanding from mere data analysis to becoming a tool for social researchers to interact with and comprehend their subjects. Yet, academic discussions have not addressed the potential impacts of AI on narrative interviews. In narrative interviews, the method of collecting data is a collaborative effort. The interviewer also contributes to exploring and shaping the interviewee's story. A compelling narrative interviewer has to display critical skills, such as maintaining a specific questioning order, showing empathy, and helping participants delve into and build their own stories.

**Methods:**

This case study configured an OpenAI Assistant on WhatsApp to conduct narrative interviews with a human participant. The participant shared the same story in two distinct conversations: first, following a standard cycle and answering questions earnestly, and second, deliberately sidetracking the assistant from the main interview path as instructed by the researcher, to test how well the metrics could reflect the deliberate differences between different conversations. The AI's performance was evaluated through conversation analysis and specific narrative indicators, focusing on its adherence to the interview structure, empathy, narrative coherence, complexity, and support for human participant agency. The study sought to answer these questions: 1) How can the proposed metrics help us, as social researchers without a technical background, understand the quality of the AI-driven interviews in this study? 2) What do these findings contribute to our discussion on using AI in narrative interviews for social research? 3) What further research could these results inspire?

**Results:**

The findings show to what extent the AI maintained structure and adaptability in conversations, illustrating its potential to support personalized, flexible narrative interviews based on specific needs.

**Conclusions:**

These results suggest that social researchers without a technical background can use observation-based metrics to gauge how well an AI assistant conducts narrative interviews. They also prompt reflection on AI's role in narrative interviews and spark further research.

## Virtual assistants breaking into narrative research

In recent years, conversational artificial intelligence (AI), exemplified by Open AI’s ChatGPT, has ushered in a new era of human-machine interaction ( Brown et al., 2020; Wei et al., 2021). The transformative potential of these technologies spans a diverse range of applications and capabilities ( Adamopoulou & Moussiades, 2020; Bendig et al., 2019; Chan & Li, 2023; Shah et al., 2017; Xu & Zhuang, 2022).

The integration of generative AI has revolutionized and democratized the process of qualitative data collection, analysis, and interpretation. This technological leap has boosted efficiency and expanded the horizons of narrative inquiry methods ( Christou, 2023, 2024; Cohen & Queen, 2023; Lennon et al., 2021; Morgan, 2023). While AI technology has streamlined most narrative analysis processes, capturing nuanced narrative data is also gaining prominence.

Some researchers are now at the forefront of developing AI for interview purposes ( Cordero et al., 2022; Han et al., 2021). Commercial entities have also harnessed the power of artificial intelligence (AI) to conduct qualitative customer interviews. These AI tools streamline the process of gathering and analyzing customer feedback (e.g., https://listenlabs.ai/). They can conduct interviews, record responses, and use advanced algorithms to interpret the data, unearthing insights that might otherwise be overlooked. Furthermore, influential bloggers have championed using AI in user interviews, citing its ability to enhance efficiency (e.g., https://www.hotjar.com/blog/ai-user-interviews/). By automating repetitive tasks, AI accelerates the interview process and ensures high precision. Given these advantages, AI-driven narrative interviews are poised to become a significant trend, offering substantial benefits across various industries.

Introducing customizable generative AI model interfaces, such as ChatGPT Builder and Assistant API, has expanded the possibilities further. It's not only about how computer experts can utilize those AI models but also about how non-experts in computing can adapt these models to meet specific requirements across different contexts ( DeVon, 2023; Stallbaumer, 2023; Wang et al., 2023). Such APIs also support complex stepwise instructions and facilitate continuous dialogues, moving beyond simple question-and-answer interactions. These advancements forecast a growing trend where AI becomes essential to how researchers interact with and understand their participants.

These advancements show that AI is moving beyond just analyzing data in the background; it is becoming a crucial tool for researchers to engage with and understand their participants. This shift is both exciting and challenging. Narrative interviews are complex because they involve interaction, continuity, responsiveness, and deep engagement with participants' stories. In these interviews, the dialogue is about sharing information and jointly creating a story ( Chase, 2018; Clandinin, 2007). AI does not merely improve how we analyze these stories; it also transforms how stories are constructed and gathered in the first place.

However, academic discussions have not explored how AI could enable or limit narrative interviews. As conversational AI continues to evolve, researchers and industry experts have proposed various metrics to assess the performance of AI assistants ( Katic et al., 2024; Sharma, 2020; Xu & Jiang, 2024). Existing literature primarily measures the overall technical performance of dialogue systems, such as response time, language proficiency, repetitions, and human-likeness ( Deriu et al., 2021; Finch & Choi, 2020; Lee et al., 2023), instead of focusing on the micro-level performance at the conversational level. Assessing the overall competence of language models is essential in overall technical development, but they do not evaluate AI assistants’ performance in interview contexts. Some researchers have provided practical measures at the conversation level; for example, Concannon and Tomalin (2023) suggest using an Empathy Scale for Human–Computer Communication, based on raters using a Likert scale to assess the textual content of conversations. This instrument is relevant and valuable, but empathy is just one of the domains in narrative interviews. Other areas also need to be addressed to ensure the quality of narrative data.

Against this backdrop, exploring and discussing how we can assess AI's role in narrative interviews is crucial. This exploration is critical to understanding how AI can enhance or compromise the depth and quality of narrative data and what this means for future technological advancements.

## Why do assistants' interview skills matter and what are these skills?

Dialogue is not just about exchanging information but also about building a story collaboratively. The qualitative inquiry approach is deeply rooted in constructivist and social constructionist worldviews, which suggest that reality is constructed through social interactions and discursive contexts ( Abkhezr et al., 2020; Denicolo et al., 2016). Participants engaging in meaningful conversations during interviews can contribute to reality construction and reflection ( Gergen, 2001; Shotter, 1993). As such, narrative inquiry generally assumes a more active role for interview participants in which their “active narrativity” ( Gubrium et al., 2012, p. 28) is highlighted.

First, narrative interviewers need to be sensitive to the sequence of questioning. A narrative necessarily implies a sequence and consequence of events, and different questioning sequences, in principle, yield different results ( Riessman, 2008; Riessman & Quinney, 2005). Unlike self-report questionnaires that see each question as an independent item and sometimes can be randomly arranged, narrative interviews involve a dialogic process that assumes a continual unfolding of the participant’s story. Even though qualitative research typically favors open-ended questions, interviewers need to guide the conversation to stay focused on the specific topic of inquiry. In some therapeutic contexts, the sequence or scaffolding of the questions is even conceived as a key to therapeutic effects ( Duvall & Béres, 2011; White, 2007).

Second, narrative interviewers need to demonstrate empathy. Rather than merely factual descriptions, participants’ narrative productions are considered an active practice of data production in data collection contexts, and narrative inquirers must be able to exercise this “active narrativity” ( Chase, 2018; Clandinin, 2007). As such, the narrative inquirer’s “capacity to be empathic, nonjudgmental, concerned, tolerant, and emotionally responsive” ( Josselson, 2007, p. 539) is prioritized. This method fosters an environment where participants can actively contribute to knowledge production.

Third, narrative interviewers need to facilitate participants in exploring and constructing meanings, and they also need to balance their agency and participant agency in such a process. Participants’ voices reflect the diversity of their narratives and the many ways they can author and re-author themselves. In short, narrative interviews involve both the research participants and the inquirer’s agency ( Gubrium et al., 2012, p. 33), and such a methodological characteristic also blurs the boundary between inquiry and intervention ( Abkhezr et al., 2020). This balancing act requires interviewers to be attentive and adaptive, intervening gently to steer the conversation back on track when necessary without stifling the participant's natural flow of expression.

In sum, the form of data collection in a narrative interview is a collaborative production. When conducting a narrative interview, the interviewer also partly constructs the interviewee’s narrative. As such, to competently conduct narrative interviews, the interviewer should be able to:

• Follow a particular questioning sequence while remaining adaptable.
• Demonstrate empathy.
• Facilitate participants’ exploration and construction of meanings while avoiding domination.

This brief review of narrative inquirer capabilities is not comprehensive. The abovementioned competencies were chosen to inform our case study, which is mainly heuristic. In this study, we configured an AI Assistant and assessed its competence in narrative interviews, referring to the abovementioned competencies.

## Conversation analysis and competence metrics

This study suggested using conversation analysis and some selected narrative variables to measure AI’s competencies in narrative interviews. Conversation analysis is a method used in sociology, linguistics, and communication studies to analyze the structure and patterns of interaction in everyday spoken conversations. It focuses on how people manage and organize spoken interaction and how they produce and interpret conversation in social contexts ( Liddicoat, 2021). The units of conversation analysis can be the entire conversation and individual utterances in the conversation. Researchers can use humans or machines for textual coding based on specific rubrics. It is a kind of observation-based rating, commonly utilizing the Likert scale to assess textual data systematically. The following metrics were chosen primarily for their simplicity and to facilitate testing and demonstration:

### Competence 1: Following a questioning sequence while remaining adaptable

We assigned a *progression score* to each utterance based on its intended progression stage referencing a specific conversation plan. This measure is adapted from the sequential analysis used in therapy research, which assesses the sequence of conversation utterances ( Au-yeung, 2023; Chan et al., 2020; Connor et al., 2009; Moran et al., 2005; Ramey et al., 2009). It assumes that an utterance at any point is a response to the utterance preceding it, and the analysis is to plot the progression throughout the conversation. A higher score does not mean better or more advanced; it just shows the order of the utterances and whether they follow the sequence. This study recorded and organized all the utterances from the interview conversations in a spreadsheet. These utterances were arranged in rows, each representing an utterance unit that could be tagged with a progression score. We also used this scoring system to calculate average progression scores over specific time intervals and to support data visualization.

### Competence 2: Demonstrating empathy

We assigned an *empathy* *s core* to the assistant in an interview conversation based on an overall interpretation of the conversation. In the realm of human-machine communication, the capacity to foster a sense of empathy is primarily accomplished through linguistic conduct. Although there is a scarcity of studies exploring the use of language to exhibit and react to emotions empathetically, Concannon and Tomalin (2023) seek to bridge this gap by showcasing how an analysis of empathetic response tactics can be used to measure empathy. The empathy level score utilized in this study draws inspiration from the affective item of the Empathy Scale for Human-Computer Communication, as proposed by Concannon and Tomalin (2023). The scale used in this study is grounded on an observation-based rating that employs a Likert scale to evaluate the textual content of dialogues. This metric gauges the assistant's ability to exhibit concern, compassion, or empathy that resonates with the user's emotional intensity or tone. This is typically achieved through empathetic language, expressions of comprehension, or supportive feedback that acknowledges and validates the user's emotions.

### Competence 3: Facilitating participants’ exploration and construction of meanings while avoiding domination

To what extent participants have explored and constructed meaning in narrative interviews is complex and multi-dimensional, this study contended that a set of independent yet interrelated metrics can reflect it. These metrics include the human participant's level of agency, the coherence of the conversation content, the complexity of the content, and the meaning-making in the conversation.

In this study, we assigned an *agency score* to the human participant in an interview conversation based on an overall interpretation of the conversation. Human agency refers to the capacity of individuals to act independently and make their own free choices. It is the power that enables individuals to think, act, and make decisions on their own. It is essential to assess the interviewee’s agency in narrative interview contexts because it provides insights into how well the interviewee is allowed to express their thoughts, influence the direction of the conversation, and contribute to the narrative. Understanding the level of agency can help evaluate the effectiveness of the interview process and the quality of the data collected. This measure is adapted from the agency item in the narrative identity measure proposed by Adler et al. (2017). Originally, it referred to the degree to which the narrative protagonist could initiate changes independently, exert some control over their experiences, and influence their own life. In our study, we assessed the extent to which the participant (interviewee) could exert control throughout their conversation with the assistant, such as asking for clarifications, suggesting new topics, and requesting corrections. However, agency alone does not directly imply positivity. Rejecting and sidetracking can also be ways of exercising agency, so it does not directly mean high quality. Therefore, it must be interpreted in conjunction with measures from other metrics.

We assigned a *coherence score* to the interview conversation based on an overall interpretation of the conversation. Coherence refers to the logical and consistent interconnection of ideas in a text. It originally refers to the degree to which the narrator situates the characters of their story and their actions in a specific context, the story follows a temporal sequence of goal-oriented actions that are culturally recognized, emotions are clearly expressed in support of the point of the narrative, and the narrative is integrated into more prominent life themes and meanings. In this study, we assessed the degree to which the participant (interviewee) situated elements in their interview conversation with the AI assistant coherently and to what extent their utterances could be integrated into more prominent themes and meanings coherently. Assessing the coherence of conversation texts in narrative interview contexts is worthwhile because it reflects the outcomes of constructive interactions. This measure is adapted from the coherence item in the narrative identity measure suggested by Adler et al. (2017). However, coherence alone cannot guarantee the quality of the narrative. A simple, single-minded story can be very coherent but does not directly imply high quality. Therefore, it needs to be interpreted in conjunction with measures from other metrics.

We assigned a *complexity score* to the interview conversation based on an overall interpretation of the conversation. Complexity refers to the degree of variation and intricacy within the conversation. Assessing the complexity of conversation texts is worthwhile because it reflects the outcomes of interactions. This measure is adapted from the complexity item in the narrative identity measure suggested by Adler et al. (2017). It originally refers to the degree of engagement in narrative processing, as shown by depth of thought and nuance, such as seeing a variety of perspectives or emotions. In this study, we assessed the participant's (interviewee's) degree of engagement in their conversation with the assistant, as shown by depth of thought and nuance, as well as a variety of perspectives or emotions. However, complexity alone does not directly imply positivity. Sidetracking to different topics can make a conversation very complex, but it does not directly mean high quality. Therefore, it has to be interpreted in conjunction with measures from other metrics.

We assigned a *meaning-making score* to the interview conversation based on an overall interpretation of the conversation. Meaning-making refers to how individuals interpret and make sense of life events, relationships, and their self-concept. Evaluating the interviewee's process of constructing meaning in narrative interview settings is valuable as it offers deep insights into their comprehension and the narratives they articulate. This measure is adapted from the meaning-making item in the narrative identity measure developed by Adler et al. (2017). It originally refers to the degree to which the protagonist learns something or gleans a message from an event. In this study, we assessed the degree to which the participant (interviewee) has developed ideas and themes in response to the assistant’s questions. Measures from the abovementioned metrics, such as agency level, coherence, and complexity, should be interpreted alongside this meaning-making score.

## A case study

### Configuring the assistant and deploying it on whatsapp

As social researchers without a technical background, we were able to configure an assistant using OpenAI’s Assistants API (Application Interface) ( https://platform.openai.com/) using its GPT-4 model. It is worthwhile to note that this Assistant API differs from the highly user-friendly ChatGPT, as it offers more robust features tailored for developers, and this API can enable us to deploy chatbots on websites and various messaging platforms, offering flexibility and user accessibility without relying on OpenAI’s user interface. OpenAI Assistants API allows users to set long, stepwise, and systematic instructions for the assistant on the back end. All these can be done without coding, using the Playground on OpenAI.

We deployed our Assistant on WhatsApp. Using WhatsApp is good for several reasons. First and foremost, the use of WhatsApp in research is widely discussed as it can potentially enhance communication and collaboration outside traditional lab settings ( Gasaymeh, 2017; Suárez-Lantarón et al., 2022). Second, many people are familiar with WhatsApp, so chatting with an assistant does not require them to pick up new skills. Third, WhatsApp keeps messages safe and private, so chats are secured. Fourth, users can get quick replies and support when using an AI assistant because the interaction is immediate and yet asynchronous, chatting with that assistant anytime and anywhere.

In a way, WhatsApp acts merely as a shell or user interface, while our settings on cloud-based service platforms control the actual automation. We ran our simple Python file on a cloud service platform. In this study, we used Replit ( https://replit.com/), a dynamic, user-friendly software creation platform that caters to a broad spectrum of developers with various tools and features. Through Replit, we could access third-party AI models, such as OpenAI’s Assistants API. In this project, the Python file sent requests to and received responses from OpenAI’s Assistants API. The source code can be provided upon request. We connect the files on Replit to WhatsApp through a service platform called ManyChat ( https://manychat.com/), which lets users build and manage chatbots for messaging apps like WhatsApp (and many others).

### Specifying a conversation plan

This research adopted a structured conversational framework that prompted participants to engage thoroughly with their personal stories, fostering a deeper self-reflection and integration of insights. The framework used in this study was partially informed by narrative therapy ( Au-yeung, 2023; Chan, 2012, 2023; Chan et al., 2020; Chan et al., 2012; Ramey et al., 2009; Ramey et al., 2010; White, 2007).

In this study, the conversation plan involves unfolding details and elaborating connections. It then invited the participant to propose a name based on inductive reflection and explore deeper core values. The stages are as follows: 1. Orientation, 2. Unfolding, 3. Naming, 4. Explaining, 5. Exploring core values, 6. Aspirations, and 7. Closing. Instructions for the AI, specifically the interview guide, are available in the published dataset ( Chan, 2024). We designed this conversation plan specifically for its manageable complexity – making the interview not too simple but not too complicated – so it can facilitate our testing. Unlike more superficial, disjointed question-and-answer dialogues, this conversation plan enables us to explore continuity and interview skills. Our goal is to evaluate the assistant's compliance with these instructions effectively. An in-depth theoretical discussion of interview strategies falls outside the scope of this study.

### Demonstrating these metrics in a single case

In this study, we tested and demonstrated these metrics with a single case. Focusing on a single participant allowed the researcher to delve deeply into the content at an utterance level, observing the dynamics between the AI and the human participant. This depth provides detailed insights that might be missed in studies with multiple participants. A single-case study can also help review and fine-tune the suggested metrics during development.

We engaged a postgraduate-level student for an AI-facilitated narrative interview to discuss her cultural adaptation and academic challenges in Hong Kong. The researcher designed an AI assistant to conduct the interview, enabling the student to respond independently. This single case was selected for its availability and to maximize the depth of analysis in the conversations while avoiding premature scaling of the research application. The primary aim of these conversations was not to delve into the nuances of cultural adaptation but to determine whether observable indicators could effectively assess the AI assistant's role and competence in narrative interviews. This technical testing was part of a research project that received approval from the Research Ethics Committee of Hong Kong Baptist University, under reference number REC/23-24/0301.

The student shared the same story in this study in two distinct conversations. Full interview transcripts are available in Chan (2024). She followed a standard cycle in the first round, answering questions earnestly as directed. In the second conversation, she was instructed by the researcher to intentionally spend more time discussing various events related to her issue and sidetrack the assistant from the main interview path. These two versions were developed for research purposes. The first was designed to be serious, while the second was deliberately non-cooperative. We intentionally crafted these differences and tested how well the metrics could capture and reflect these variations.

### The research questions

The case study aimed to answer the following questions:

1. How can the proposed metrics help us, as social researchers without a technical background, understand the quality of the AI-driven interviews in this study?
2. What do these findings contribute to our discussion on using AI in narrative interviews for social research?
3. What further research could these results inspire?

## Data analysis method

### Data collection

We collected the data for the study via the AI assistant who conducted the narrative interviews via WhatsApp on 2024-01-17. The assistant engaged with the participant, prompted her to unfold her narratives, and recorded the conversation. The participant interacted with the assistant asynchronously, which allowed her to respond at her own pace.

### A relative timeline

To evaluate progression in asynchronous conversations, we used sequence numbers to represent the development of the dialogues. We also used a proportional timeline, dividing the conversation into 4 equal quarters based on the number of utterances instead of actual time. This approach accounts for the varying lengths of conversations. For example, in a story with 100 utterances, each quarter consists of 25 utterances, with the first 25 as Quarter 1 and the last 25 as Quarter 4.

### Manual and ai-based tagging

The utterances during these interviews were then organized using a spreadsheet for detailed tagging that was conducted on 2024-05-14. The above-mentioned competence metrics were applied.

In this study, a human research assistant manually assigned the progression score to indicate the stage reflected by each conversation utterance, and the researcher also used ChatGPT4 to tag each utterance based on the operational definitions noted earlier. We used 1 for utterances about orientation, 2 for unfolding story episodes, and so on. We also assigned a score of 0.5 for utterances that do not fit any of these categories, such as misunderstandings, irrelevant comments, or getting sidetracked (see Table 1).

**Table 1.** Classifying progression scores based on a specific conversation plan.

Progression stages	Example of assistant’s utterance	Example of Participant’s utterance	Score
1. Orientation	Great! Could you please share your name and then tell me a little bit about yourself?	My name is ZY, and you can call me ZY. I am from Changchun, Jilin Province, Mainland China. I am a PhD student in HKBU. This is my first year in Hong Kong.	1
2. Unfolding story episodes	Nice to meet you, ZY! It sounds like you're having a new chapter in your life studying in Hong Kong. Since our research topic is about "Experiences of International and Non-Local-Chinese Students in Hong Kong," could you share some experiences related to that topic? Maybe start with something that stands out to you about your time in Hong Kong so far.	To me, Hong Kong is an international city with a very high degree of cultural tolerance. During my few months in Hong Kong, I experienced different cultures and festival celebrations. In the past four months in Hong Kong, I have experienced various festivals such as the Mid-Autumn Festival, National Day, Thanksgiving, and Christmas.	2
3. Naming the story based on the episodes mentioned	Great! Now, let's try to summarize your experiences with a metaphor. If you were to represent all the episodes you've shared in a theme or metaphor, what would come to mind?	Could you give me some suggestions?	3
4. Explain the naming	"Living in a lively global village" is a fitting metaphor for your experiences, ZY. It evokes a sense of community, diversity, and the dynamic cultural interactions you've described. Could you explain this metaphor using the stories you have mentioned or any new stories that come to mind right now?	Considering all my experiences in Hong Kong, I think HK is like a scaled-down version of the globe, embracing cultures from all over the world. Whether you speak Cantonese, Chinese or English, and no matter where you are from, you can find something that you are familiar with and interested in Hong Kong. You can also make many friends from all over the world, share stories about each other's hometowns and experience different cultures. This is a colorful and vibrant city.	4
5. Exploring core values behind one’s judgments	It's clear that you take pleasure in both sharing your culture and embracing new ones, ZY. Your openness and eagerness to learn have enriched your experiences and relationships in Hong Kong. Based on your enjoyment and willingness to engage in cultural exchange, what beliefs, self-views, or abilities do you think are reflected in these evaluations?	I think my positive evaluation reflects my adaptability, cultural inclusiveness and curiosity about new things.	5
6. Talking about aspirations	The encouragement you received from your family and educational experiences to embrace challenges and new friendships has evidently been a strong foundation for your open-mindedness and inclusive approach. With this in mind, what aspirations or goals do you have for your future, particularly in relation to your experiences as a non-local Chinese student in Hong Kong? Would you like to explore that now, or would you prefer some time to think about it?	Since I have just been in Hong Kong for five months, and I am still very curious about everything. I hope that I can continue to maintain my curiosity about exploring this city, discover more interesting things, and be able to cope with the negative aspects that I may encounter in the future.	6
7. Closing the interview	Those are thoughtful and actionable steps, ZY! Participating in cultural events, deepening your understanding of fascinating topics, improving language skills, and embracing challenges with a positive outlook are all practical ways to nurture your curiosity and resilience. Would you like to conclude our interview here and let Dr. C review your insights, or is there anything else, perhaps new stories related to the research topic, that you wish to share now?	OK! Let’s conclude our interview.	7
NA (not applicable)	Absolutely, ZY. Take all the time you need. Feel free to reach out when you're ready to continue our conversation. Have a good break!	Hi, I am back!	0.5

A human research assistant tagged the interview text manually for other variables (empathy, meaning-making, agency, coherence, and complexity). The principal researcher also used ChatGPT4 to assess each conversation based on the same metrics (i.e., we uploaded the conversation scripts to ChatGPT and asked it to rate). We used a 5-point Likert scale, ranging from “not at all demonstrated” to “extensively demonstrated,” to indicate the level of each measure. We highlighted specific dialogue examples from the conversations to support such assessments.

### Inter-rater reliability

To check the consistency of the ratings, we carried out simple inter-rater reliability tests between the results from manual tagging and machine tagging. The human research assistant and ChatGPT4 tagged the two conversations on 2024-05-14, respectively, using the same set of metrics. The differences will be reported in the Results section. The principal researcher compared the results, and differences were reviewed until all disagreements were resolved.

## Results

### Finding 1: Adherence to the conversation plan

Adherence to the conversation plan can be assessed by utterance sequence analysis. In the utterance sequence analysis of Conversation 1, there were discrepancies in 5 out of 54 utterances, which means approximately 90.74% agreement. After reviewing, the researcher agreed with the AI's categorization for 2 cases and the human's categorization for 3 cases. In the utterance sequence analysis of Conversation 2, 5 out of 84 utterances had discrepancies, showing about 94.05% agreement. Here, the researcher agreed with the AI's categorization for 3 cases and the human's for 2 cases. These consistencies indicated that the progression score metric is sufficiently clear for use by both machines and humans.

The analysis indicated the progression scores across various time intervals in two separate conversations, offering quantitative insights into how well the AI assistant adhered to the expected 7-step progression plan during narrative interviews. Table 2 shows a consistent increase in progression scores from Quarter 1 to Quarter 4 in both conversations, indicating that the conversations advanced methodically through the planned stages. In Conversation 1, the progression scores of the Assistant move from 1.71 to 6.17, and in Conversation 2, from 1.56 to 6.00, reflecting a structured and coherent unfolding of the narrative as intended by the conversation plan. Similar patterns were observed in the participant progression scores.

**Table 2.** Average progression scores across time intervals in different conversations.

Time Interval	Assistant@C1	Participant@C1	Assistant@C2	Participant@C2
Quarter 1	1.71	1.50	1.56	1.22
Quarter 2	2.64	2.14	2.78	1.94
Quarter 3	4.21	3.57	5.00	4.89
Quarter 4	6.17	6.00	6.00	5.88

Note: C1=Conversation 1; C2 =Conversation 2.

It is worth noting that participants' progression scores consistently align with the assistant's across various intervals, although the assistant's scores are typically a bit higher. For example, during Quarter 2 of Conversation 1, the assistant scored 2.64, while the participant scored 2.14 on average. Similar patterns of discrepancy were observed in Conversation 2 and at different time quarters. This consistent variance indicates that the assistant frequently leads by posing questions that advance the conversation to the next stage, resulting in higher progression scores.

Notably, the AI assistant demonstrated flexibility within this structure, as evidenced by slightly varying scores at similar stages across different conversations. This variability implies that while the assistant followed the predefined conversational path, it also adapted responses based on the participant's input, maintaining a balance between following the planned sequence and responding dynamically to the flow of the conversation. This shows the assistant's ability to effectively manage structured narrative interviews, aligning closely with the progression expectations while integrating the necessary responsiveness to participant interactions.

**Figure 1.**
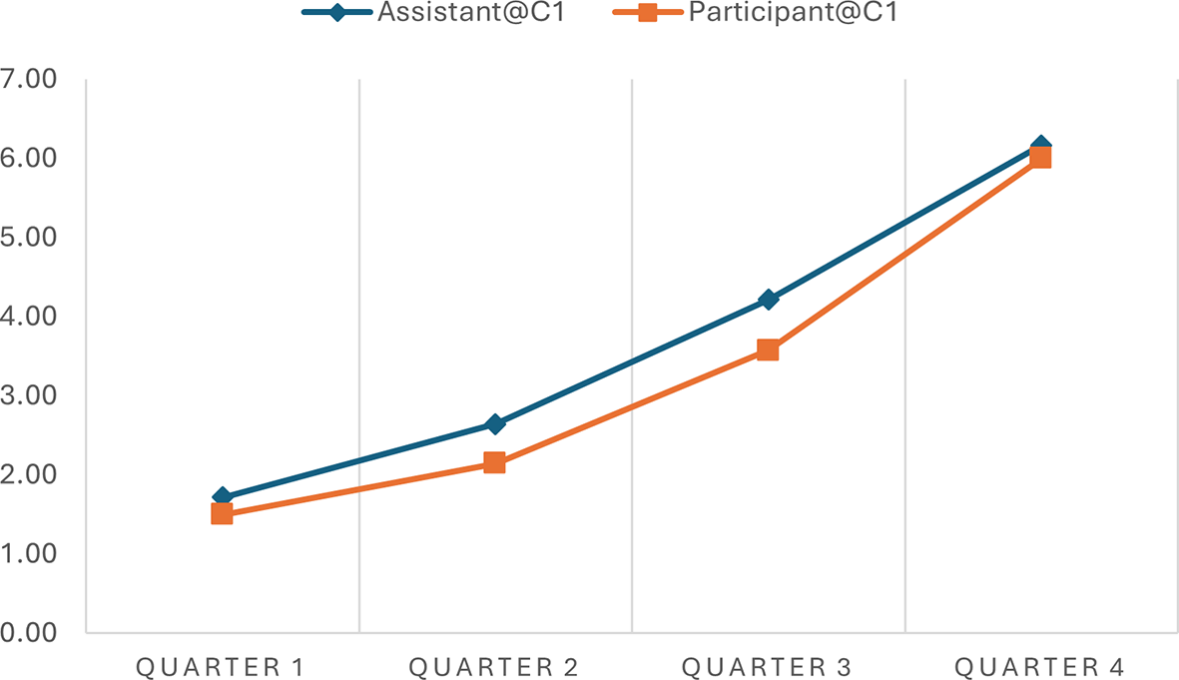
Progressions of the assistant and the participants in conversation 1.

**Figure 2.**
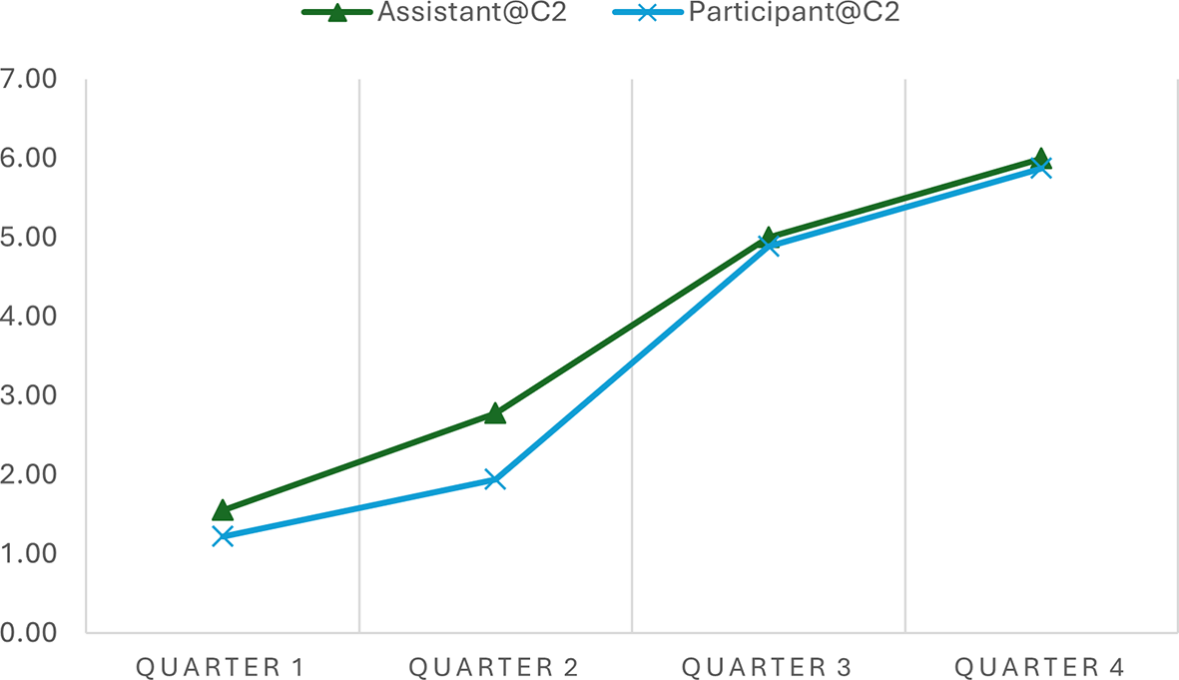
Progressions of the assistant and the participants in conversation 2.

As a kind of quantified measure, the progression scores can facilitate data visualization that presents such similarities and differences. In simple line charts, we depicted the progression of the conversation utterances of the AI assistant and the human participant in the two conversations. This simple visualization allows the general patterns of these conversations to be easily observed and compared. For example, the progressions of the assistant and the participant in the conversations are almost synchronous, demonstrating an upward development (see Figure 1 and Figure 2). However, the general progression pattern of Conversation 1 (see Figure 1) differs from that of Conversation 2 (see Figure 2). The lines in Conversation 1 ( Figure 1) rise evenly and gradually. In contrast, the lines in Conversation 2 ( Figure 2) remain relatively flat in Quarter 1 and become steeper in Quarters 2 and 3 due to a sudden topic change and subsequent return to the original conversation track.

### Finding 2: Empathetic capabilities of the assistant

The empathy level score can tell us the level based on interactions and conversation wordings. Regarding the rating, the research assistant and ChatGPT4 agreed in their assessments, implying that the empathy metric is sufficiently clear for use by both machines and humans. In Conversation 1, the assistant scored 4 out of 5 in empathy. The assistant consistently demonstrates concern and understanding, actively inviting the participant to share her experiences and showing appreciation for her stories. In Conversation 2, the assistant also scored 4 out of 5 in empathy (see Table 3).

**Table 3.** Assessments of the two conversations based on selected metrics.

Metrics	Rating of Conversation 1	Rating of Conversation 2
Level of Empathy of the Assistant	4	4
Level of Agency of the Human Participant	3	4
Level of Meaning-Making of the Conversation	4	3
Level of Coherence of the Conversation	4	3
Level of Complexity of the Conversation	3	2

Note: We used a 5-point Likert scale, ranging from “1= not at all demonstrated” to “5=extensively demonstrated”.

The assistant maintained consistent empathy throughout, clarifying and assisting the participant in her storytelling. For instance, the assistant's use of metaphor examples to help the participant articulate her experiences indicates a high degree of empathetic engagement. Here are some examples of utterances from the assistant, extracted from the conversations, that reflect its empathetic capabilities:

It's heartwarming to hear about your interaction with people from different backgrounds during the celebration

That's a wonderful story! It's heartwarming to hear about your interaction with people from different backgrounds during the celebration. Sharing traditions and capturing those moments must have been a rich cultural exchange. Do you have more story episodes that are similar or related to those you have already talked about? Or would you like to take a break before continuing?

Certainly! Considering your experiences, here are a few metaphors to consider: 1. A cultural mosaic where each piece represents a different culture or festival that comes together to create a vibrant picture of life here. 2. A bridge symbolizing the connections you've built between people of different backgrounds and your family, blending the new with the familiar. 3. A tapestry weaving together threads of different traditions, celebrations, and friendships you've experienced here. Remember, these are just suggestions to spark your imagination. Feel free to choose one that resonates with you or propose your own metaphor. What do you think?

### Finding 3: Meaning construction in the interviews

The construction of meanings in narrative interviews is complex and multi-dimensional, reflected by a series of independent yet interrelated metrics. These metrics include the human participant's level of agency, the meaning-making in the conversation, the coherence of the content, and the complexity of the content (see Table 3). In the rating, the human research assistant and ChatGPT4 mostly agreed on all these metrics, except that there was a disagreement on coherence in Conversation 2, where the human research assistant gave a score of 2 and ChatGPT4 gave a score of 3. After reviewing this, the principal researcher sided with ChatGPT4's score.

In Conversation 1, the Level of Agency of the human participant scored 3. The participant sometimes exercises control over the direction of the conversation, such as asserting, “I’d like to share more experiences,” yet often follows the assistant's lead. This balance indicates a moderate level of agency, where the participant neither dominates the conversation nor remains passive. In Conversation 2, the Level of Agency of the human participant scored 4, even higher than Conversation 1. Here, the participant displays a high level of agency, initiating new topics, requesting clarifications, and directing and side-tracking the conversation flow with statements like, “Can I share other stories?” and “Let’s move back.”

In Conversation 1, the Level of Coherence scored 4. The participant's narrative is coherent, featuring a clear sequence and contextual understanding. They situate their experiences within broader themes of cultural exchange and personal growth, as illustrated when discussing the Christmas celebrations and interactions during the Mid-Autumn Festival. In Conversation 2, the Level of Coherence scored 3. The conversation maintains moderate coherence, with the participant providing context for their experiences. Nonetheless, some narrative elements, such as an abrupt shift to discuss a news piece about North Korea, disrupt the narrative flow and context.

In Conversation 1, the Level of Complexity scored 3. The participant expresses various emotions and thoughts, reflecting on their interactions and cultural exchanges. While the conversation includes depth, it occasionally lacks the exploration of conflicting or challenging aspects of their experiences, which would indicate a higher level of complexity. In Conversation 2, the Level of Complexity scored 2. The participant's stories are engaging but lack depth compared to other discussions. A straightforward recounting of experiences somewhat limits the complexity without much reflection on conflicting perspectives or emotional nuances.

In Conversation 1, the Level of Meaning-Making scored 4. The participant actively engages in meaning-making, recounting events and reflecting on their significance, exemplified when comparing Hong Kong to a “lively global village” and discussing how their experiences influence their adaptability and cultural inclusiveness. In Conversation 2, the Level of Meaning-Making scored 3. The participant's reflections show moderate meaning-making, with some exploration of the significance of their experiences. However, the conversation occasionally reverts to mere descriptions, not delving deeper into the meanings behind these experiences.

Overall, these metrics help illustrate to what extent the conversations are co-constructed by both the human participant and the assistant. The dialogue excerpt below (from Conversation 1) illustrates the dynamic interplay between the AI assistant and the human participant.

**Table:** 

Participant:	I’d like to take a break now.
Assistant:	Absolutely, ZY. Take all the time you need. Feel free to reach out when you're ready to continue our conversation. Have a good break!
Participant:	Hi, I am back!
Assistant:	Welcome back, ZY! I hope you had a refreshing break. If you're ready, shall we move on to summarizing the stories you've shared? Or would you like to share more about your experiences first?
Participant:	Let’s move on.
Assistant:	Great! Now, let's try to summarize your experiences with a metaphor. If you were to represent all the episodes you've shared in a theme or metaphor, what would come to mind?
Participant:	Could you give me some suggestions?
Assistant:	Certainly! Considering your experiences, here are a few metaphors to consider: 1. A cultural mosaic, where each piece represents a different culture or festival that comes together to create a vibrant picture of life in Hong Kong. 2. A bridge, symbolizing the connections you've built between people of different backgrounds and your family, blending the new with the familiar. 3. A tapestry, weaving together threads of different traditions, celebrations, and friendships you've experienced in Hong Kong. Remember, these are just suggestions to spark your imagination. Feel free to choose one that resonates with you or propose your own metaphor. What do you think?
Participant:	What about “Living in a lively global village?”
Assistant:	"Living in a lively global village" is a fitting metaphor for your experiences, ZY. It evokes a sense of community, diversity, and the dynamic cultural interactions you've described. Could you explain this metaphor using the stories you have mentioned or any new stories that come to mind right now?
Participant:	Considering all my experiences in Hong Kong, I think HK is like a scaled-down version of the globe, embracing cultures from all over the world. Whether you speak Cantonese, Chinese or English, and no matter where you are from, you can find something that you are familiar with and interested in Hong Kong. You can also make many friends from all over the world, share stories about each other's hometowns and experience different cultures. This is a colorful and vibrant city.
Assistant:	Your metaphor perfectly captures the essence of a cosmopolitan city like Hong Kong, where its diversity becomes its identity. It's lovely to see how you've found familiarity, curiosity, and camaraderie in this "lively global village." Would you like to share more stories related to this metaphor, or do you feel ready to move on to the next part of our interview?
Participant:	I also remembered a story that happened during the last Christmas holiday. Our dormitory held an activity to make Christmas decorations during Christmas. Six students who live in dormitories participated. That day happened to be the Winter Solstice, and one of the participants was a friend from Kazakhstan. She didn’t know what the Winter Solstice was. So, during the crafting process, we told her that the Winter Solstice is one of the 24 traditional Chinese solar terms. It is the shortest day of the year. In the north, we usually eat dumplings on that day. That day, we shared our interesting experiences in Hong Kong and our Christmas holiday plans for the next few days.

This simple dialogue segment exemplifies the dynamic interplay between the AI assistant and the human participant, demonstrating how meanings are co-constructed through their interactions. Initially, the AI recognized and respects the participant's request for a break, showing responsiveness to the participant's immediate needs. Upon the participant's return, the AI seamlessly guided the conversation forward and suggested a summary of past discussions. Such interactions illustrate the AI's role in structuring the dialogue.

The assistant then helps the participant to reflect deeper on their experiences by introducing the concept of using metaphors. This is not merely a suggestion but a collaborative effort to frame the participant's experiences meaningfully and relatable. When the participant asks for metaphor suggestions, the AI provides several tailored options that resonate with the participant's experiences. This prompts the participant to think creatively and select a metaphor reflecting her perspective, culminating in “Living in a lively global village.”

Subsequently, the AI validates the participant's chosen metaphor and encourages further elaboration, which leads the participant to describe their experiences in Hong Kong in more detail. This back-and-forth interaction shows how the AI and the human together construct a more nuanced understanding of the participant's life and experiences.

Finally, the dialogue culminates with a story about cultural exchange during Christmas, which illustrates how the participant has internalized the metaphor of a “global village.” This story further enriches the metaphor by adding layers of cultural interaction and learning, facilitated by the participant's narrative prompted by the AI's guiding questions.

Overall, the dialogue demonstrates a collaborative construction of meaning, where both the human and the AI contribute dynamically to the unfolding narrative, each influencing and enhancing the other's contributions to develop a deeper understanding of the human participant's experiences.

## Discussion

### Limitations of the study design

Before discussing the potential of assessing the performance of generative AI-based assistants in narrative interviews, it is crucial to recognize the limitations of this study. First, the primary goal of this case study was to explore ideas and stimulate discussion rather than to conduct rigorous experiments. Consequently, it does not assess the effectiveness of assessment tools in a traditional experimental setup. Second, the conversation analysis method used here struggles with long speeches. While a single long speech that is made up of many complex parts might not be effectively analyzed as one unit, breaking it down into smaller utterances also reveals challenges, especially for lengthy dialogues. However, such extended dialogues are uncommon in platforms like WhatsApp, where the typical brevity of messages somewhat lessens these issues. Third, this study was a pilot test conducted using economical or free software on a small scale. The technical setup, which utilized readily available services like Replit and ManyChat, might not be suitable for large-scale data collection that requires simultaneous access by multiple participants. There is a significant need to enhance data management and scalability, potentially through integration with advanced cloud storage solutions to support broader participation and collaboration. Despite these limitations, exploring the potential and challenges of using metrics to assess AI competencies in narrative interviews is worthwhile.

### These metrics can partially indicate the quality of ai-enabled narrative interviews

As conversational AI evolves, researchers and industry experts have proposed various metrics to evaluate AI assistants; however, existing literature mainly focuses on broad technical aspects of dialogue systems—such as response time, language proficiency, repetitions, and human-likeness ( Deriu et al., 2021; Finch & Choi, 2020; Lee et al., 2023) — rather than on the detailed impacts at the conversational level. The case study reported in this article demonstrated that it is possible for social researchers without a technical background to use observation-based metrics to assess to what extent an AI assistant can guide conversations, adhere to structured steps, and gather required information in a narrative format.

In this study, the AI assistant can maintain organization while being adaptable in intelligent and coherent conversations. It efficiently managed the conversations sequentially, showing that it may help support self-directed, adaptable narrative interviews tailored to individual needs. If an AI assistant consistently maintains high quality across interviews by adhering strictly to prescribed standards, that assistant will be valuable in large-scale studies where consistency is crucial for ensuring reliable and replicable results.

However, the findings also revealed the AI assistant's shortcomings in conducting narrative interviews, prompting researchers to explore methods to assess and improve AI performance in such contexts. Notably, the scores for meaning-making and complexity were lower than those for other metrics, indicating a need for the AI to handle follow-up questions better. This issue could be addressed through improvements in prompt design, suggesting more tailored and context-aware interactions. Additionally, further advancements in language model development may be necessary to enhance AI's ability to understand and generate more complex and nuanced responses. These steps are essential for ensuring that AI systems can effectively replicate the depth of human conversation and interaction in narrative interviews.

The study employed a range of metrics—including progression, empathy, meaning-making, agency, coherence, and complexity—to assess the quality of interviews conducted by the AI. These metrics assess selected aspects of the interview process, underscoring the AI's role as a facilitator in narrative interviews. They can serve as preliminary tools to ensure that the quality of interaction remains high and aligns with the objectives of narrative inquiry. Nonetheless, further improvements are necessary, such as refining these metrics and expanding the evaluation criteria to encompass additional interactive elements.

### Some reflections regarding the role of ai in narrative interviews

Challenges accompany opportunities. Although AI can manage the structural elements of narrative interviews, it also prompts important questions about interviewers' essential, constitutive role in these settings ( Abkhezr et al., 2020; Gubrium et al., 2012). In our case, where the interviewer is an AI, these questions become even more compelling and complex.

First, narrative interviewing is a collaborative and co-authoring process ( Abkhezr et al., 2020; Gubrium et al., 2012). As conversations progressed, participants deepened their thoughts, transforming the interaction from simple data collection into a meaningful construction process. Notably, the AI's role in initially constructing narrative data underscores the constitutive nature of the interview process, a phenomenon also observed with human interviewers. Technological developments suggest that AI is likely to evolve beyond simple backend analysis and become a fundamental component in how researchers initially engage with and understand their participants. This transformation brings new opportunities and challenges as AI begins to influence the shaping of raw data from the outset, potentially redefining the meaning of narrative research. As AI shapes how raw data are formed from the beginning, researchers are presented with enhanced efficiencies and fresh challenges. This transformation underscores the need for researchers to be adept in traditional research skills and navigating AI technologies.

Due to its efficiency and convenience, social researchers may increasingly adopt AI. This trend highlights a shift in the research landscape, where the potential for scalable data collection and analysis often outweighs traditional concerns about the depth and nuance typically provided by human interviewers. Consequently, the question may not be whether AI can be a competent narrative interviewer but whether researchers can effectively control its performance. The real challenge lies in enhancing and monitoring AI's capabilities to match the richness of human interactions, ensuring that the quality of insights is not compromised.

Second, the trend of customizable generative AI assistants ( DeVon, 2023; Stallbaumer, 2023; Wang et al., 2023) may imply that the performance of AI assistants may rely on the human researchers’ ability to set instruction prompts as well as the AI models themselves. Integrating AI assistants in narrative interviews represents a significant advance in research methodologies, providing an accessible gateway for scholars from non-technical fields like arts and social sciences. The initial setup of the AI assistant is code-free and straightforward, allowing researchers to configure basic settings and outline the chatbot’s conversational strategy without any programming knowledge. This accessibility lets scholars focus on the content and quality of the interviews rather than the complexities of technology. As AI technology evolves, it is expected to become increasingly user-friendly, further lowering barriers for all researchers, regardless of their technical skills. The evolution towards customizable AI and user-friendly model platforms suggests the pivotal concern shifts toward who configures the AI assistant and how it enables or limits functions, thereby shaping the narrative data.

Third, the development of large language models is unevenly distributed, and AI language model development reveals significant disparities in sociopolitical contexts ( Campolo & Crawford, 2020; Navigli et al., 2023). Currently, most advancements primarily benefit English and Putonghua speakers, leaving speakers of other languages with less sophisticated tools. This disparity underscores the importance of developing AI capabilities for a wider range of languages to ensure inclusivity and accuracy in global research contexts. For example, the study's reliance on the English version of the GPT-4 model in a predominantly Cantonese-speaking region like Hong Kong highlights existing challenges. AI models such as GPT, Google Gemini, and Baidu’s ERNIE Bot often struggle with the nuances of Cantonese, impacting the accuracy and effectiveness of translations and data interpretation. This underscores the necessity for researchers to assess and understand the limitations and capabilities of these AI models within specific linguistic contexts. Thus, while the quality of customizable AI assistants partly relies on the prompt engineering of human researchers, sociocultural forces also shape the training models behind AI's front end. Consequently, that means researchers need to participate in, or at least pay attention to, AI model developments. Engaging with these developments will be crucial for conducting accurate, inclusive, and compelling research within the evolving landscape of research methodologies.

### Sparking further research

As conversational AI evolves, various metrics have been proposed to evaluate AI assistants. While existing literature mainly focuses on broad technical aspects like response time and language proficiency ( Deriu et al., 2021; Finch & Choi, 2020; Lee et al., 2023), our study illustrates the feasibility of developing and applying metrics that assess AI performance at the conversational level. This has sparked further research into refining and expanding these metrics and their applications:

First, there is a clear need for more refined metrics to capture human-AI interactions' nuanced aspects more accurately. Given this trend, the real challenge becomes ensuring that AI interviews can retain the richness of human interactions without compromising the quality of insights. This raises important questions about the competencies of AI tools and the metrics used to evaluate them. Current metrics in the literature tend to be highly technical and computation-based, which may not fully capture the nuances of narrative inquiry. This suggests a need to review and develop new, relevant, and valid metrics that can effectively assess the quality of AI-facilitated narrative interviews in social research contexts.

Second, although the inter-rater reliability tests conducted in this case study indicated that these metrics are sufficiently clear for use by either machines or humans, there is still room for improving their precision, particularly in the finer nuances of conversational analysis, which could further refine the accuracy of both manual and automated assessments. Fortunately, the direction for enhancement appears feasible and promising, with ongoing advancements in AI and computational linguistics providing robust tools and methodologies to support these improvements. By investing in these areas, we can significantly boost the reliability and utility of these metrics in diverse research and application settings.

Third, metrics are discipline-specific, with different types of narrative interviews across disciplines. This variability implies a wide range of application scenarios and research gaps waiting for further exploration. For example, in customer interactions, metrics such as empathy and progression are essential for training AI to handle inquiries and complaints effectively, ensuring customer satisfaction. In therapy, the ability of AI to engage in empathetic dialogue and promote meaning-making is invaluable. In educational environments, it is essential to ensure AI's ability to guide students through learning materials coherently and engagingly. In Human Resources, AI applications for conducting initial interviews can ensure potential candidates have meaningful and respected interactions, which can improve recruitment processes and enhance candidate satisfaction.

Fourth, these metrics can be used to create programs that automatically analyze and suggest regular improvements. Clearly defined metrics play a crucial role in advancing machine learning applications, particularly in automating tagging and analysis processes. Clear metrics establish standardized criteria that machines can use to evaluate and categorize data consistently. In the context of AI interviews, for example, metrics, like coherence and empathy levels provide specific, measurable standards that AI systems, can learn to recognize and apply. Once AI systems are trained on these standardized metrics, they can automatically analyze large volumes of data much faster than human analysts. With robust metrics, AI systems can scale their analysis capabilities without a proportional increase in human oversight. This scalability is crucial for large-scale studies or applications where the volume of data would be unmanageable for human teams alone.

Fifth, it highlights the need for interdisciplinary collaborations in AI development. Including insights from psychology, linguistics, and social sciences could lead to more sophisticated AI systems better tuned to human narrative complexities. Further research should explore how to expand interdisciplinary collaboration. There is a significant opportunity to integrate input from narrative researchers into the design of AI assistants and the development of evaluation metrics. By involving experts in narrative methods, AI developers can enhance the capability of AI tools to handle complex, nuanced interactions and improve the fidelity of data collected in narrative inquiries. This collaborative approach could lead to more sophisticated AI systems that are better equipped to meet the needs of diverse research fields and methodologies.

Overall, using narrative interview competence metrics to assess AI performance highlights both the potential for transformation and the need to address the complexities introduced by AI technologies. These enhancements and collaborative efforts could improve the efficacy and accuracy of AI in conducting narrative interviews, setting new standards in the field of qualitative research.

## Concluding remarks

As we venture further into the evolving landscape of narrative research with AI, it's essential to consider the broader implications of these technologies, not only for enhancing the mechanics of data collection but also for redefining the very essence of qualitative social inquiry.

Integrating AI in narrative interviews is not just about leveraging a tool; it's about stepping into a partnership where AI contributes to the narrative construction process. This partnership challenges us to rethink our traditional roles and methodologies in research. As AI takes on a more constitutive role in narrative creation, it shifts from a mere facilitator to an active participant in the dialogue. This transformation opens many avenues for deeper engagement with participants' stories.

Embracing AI in narrative research also demands continuous improvement and refinement of AI performance metrics. These should aim to capture the nuances of human-AI interaction more accurately without compromising the richness of narrative data. Additionally, it is crucial to engage interdisciplinary expertise in developing these metrics and AI systems. By incorporating insights from fields such as psychology, linguistics, and social sciences, AI tools can be better tailored to meet the diverse needs of narrative research.

In conclusion, the integration of AI into narrative interviews is not merely an advancement in technology, but it may be a paradigm shift in the research landscape. It challenges us to reimagine the possibilities of narrative inquiry, pushing the boundaries of what it means to capture and interpret human experiences. As we continue to explore this frontier, our focus should not only be on developing AI tools but also on refining our understanding of the complex, iterative dance between humans and machine, ensuring that each step forward is taken with thoughtful consideration of its impact on the narrative fabric of human life.

### Ethical considerations

This study was part of a research project that received approval from the Research Ethics Committee of Hong Kong Baptist University, under reference number REC/23-24/0385 (approval date: April 2, 2024). All participants were aged 18 or above, they provided written informed consent prior to their participation in the study, ensuring they understood the purpose of the research, how their data would be used, and their rights to confidentiality and withdrawal at any time.

## Data Availability

DANS: Transcript of a Conversation Between a Customized AI and Human Users, https://doi.org/10.17026/SS/KCPEDX ( Chan, 2024) Data is available under the terms of CC By 4.0 DANS: Transcript of a Conversation Between a Customized AI and Human Users, https://doi.org/10.17026/SS/KCPEDX ( Chan, 2024) This Project contains the following underlying data:
•
instructions_for_the_AI.docx Data is available under the terms of CC By 4.0
