# A Case Study on Assessing AI Assistant Competence in Narrative Interviews

**DOI:** 10.12688/f1000research.151952.2

**Published:** 2024-10-04

**Authors:** Chitat Chan, Yunmeng Zhao, Jiahui Zhao

**Affiliations:** 1 Social Work, Hong Kong Baptist University, Hong Kong, Hong Kong

**Keywords:** Artificial Intelligence, Narrative Inquiry, Qualitative Research, WhatsApp Interviews, Conversational AI, Prompt Engineering, Digital Research Methodologies

## Abstract

**Background:**

Researchers are leading the development of AI designed to conduct interviews. These developments imply that AI's role is expanding from mere data analysis to becoming a tool for social researchers to interact with and comprehend their subjects. Yet, academic discussions have not addressed the potential impacts of AI on narrative interviews. In narrative interviews, the method of collecting data is a collaborative effort. The interviewer also contributes to exploring and shaping the interviewee's story. A compelling narrative interviewer has to display critical skills, such as maintaining a specific questioning order, showing empathy, and helping participants delve into and build their own stories.

**Methods:**

This case study configured an OpenAI Assistant on WhatsApp to conduct narrative interviews with a human participant. The participant shared the same story in two distinct conversations: first, following a standard cycle and answering questions earnestly, and second, deliberately sidetracking the assistant from the main interview path as instructed by the researcher, to test how well the metrics could reflect the deliberate differences between different conversations. The AI's performance was evaluated through conversation analysis and specific narrative indicators, focusing on its adherence to the interview structure, empathy, narrative coherence, complexity, and support for human participant agency. The study sought to answer these questions: 1) How can the proposed metrics help us, as social researchers without a technical background, understand the quality of the AI-driven interviews in this study? 2) What do these findings contribute to our discussion on using AI in narrative interviews for social research? 3) What further research could these results inspire?

**Results:**

The findings show to what extent the AI maintained structure and adaptability in conversations, illustrating its potential to support personalized, flexible narrative interviews based on specific needs.

**Conclusions:**

These results suggest that social researchers without a technical background can use observation-based metrics to gauge how well an AI assistant conducts narrative interviews. They also prompt reflection on AI's role in narrative interviews and spark further research.

## Democratizing AI chatbots and domain knowledge expertise

### The possibility

The use of AI chatbots in various real-life applications is rapidly increasing due to their ability to reduce reliance on humans, lower costs, improve efficiency, and streamline service experiences ( Adamopoulou & Moussiades, 2020; Bendig et al., 2019; Chan & Li, 2023; Greer et al., 2019; He et al., 2022; Liu et al., 2022; Omarov et al., 2023; Shah et al., 2017; Tamayo et al., 2020; Tanana et al., 2019; Xu & Zhuang, 2022).

Before we proceed, it is important to clarify some terminologies related to conversational user interfaces. Research on these interfaces, including dialogue systems, embodied conversational agents, and social robotics, is converging towards the development of improved conversational computing systems. These systems are increasingly referred to as “chatbots” in both industry and research, a term that broadly encompasses conversational agents designed for various purposes, such as task completion, information delivery, entertainment, and social interactions, through text, voice, or both ( Følstad et al., 2021). Unlike more narrowly defined uses of the term, this understanding of chatbots focuses on the design, development, and implications of these systems rather than specific technologies. Given the rapid evolution of technology and usage patterns, maintaining distinctions between types of conversational agents is challenging, as there often is significant overlap in functionality and modality. In this study, we generally use the term “chatbot,” but we specifically refer to ‘AI Assistant’ when discussing OpenAI’s Assistant API (Application Programming Interface).

Central to this AI chatbot proliferation is GPT (Generative Pretrained Transformer), a deep learning model that uses a self-attention mechanism to weigh different parts of input sentences and determine their importance. By converting sentences into tokens and predicting the next token’s probability, GPTs can generate coherent text that often appears human-written. Various studies have demonstrated this capability well ( Brown et al., 2020; Wei et al., 2021; Wu et al., 2021). GPT-based chatbots, for example ChatGPT, can understand natural language inputs and produce responses that are contextually appropriate and coherent, thereby improving interactivity and efficiency ( Bendig et al., 2019; Nath et al., 2021; Shah et al., 2017; Xu & Zhuang, 2022).

Since 2023, advancements in customizable features have further transformed the landscape of GPT applications, enabling not just programmers but also non-technical users to become GPT designers rather than merely end-users, thanks to simplified coding and prompt engineering ( Stallbaumer, 2023; UC Today, 2023). For example, OpenAI introduced ChatGPT Builder and Assistant API (Application Interface) in November 2023, enabling users to build custom GPTs without technical expertise or coding experience ( DeVon, 2023; UC Today, 2023). ChatGPT Builder provides a user-friendly interface for creating custom chatbot versions without coding, featuring built-in tools that OpenAI fully hosts. However, it does not support integration with other application platforms. On the other hand, the Assistant API also offers a Playground interface for building custom chatbots without coding. Unlike ChatGPT Builder, it allows developers to integrate these chatbots into various applications, such as instant messaging platforms like WhatsApp or Facebook Messenger, provided they have some coding skills and knowledge of these third-party platforms.

These backend platforms allow users to use natural language instructions to define the AI’s role and context, set its tone and behavior, and design stepwise procedures, making the chatbot’s responses relevant and coherent. For example, a customer service chatbot can be instructed to greet users politely, answer frequently asked questions, and escalate issues to a human agent if it cannot provide a satisfactory answer. Additionally, a chatbot for educational purposes can be guided to explain concepts in simple terms, provide examples, and quiz users to reinforce learning. By tailoring the AI’s behavior and responses, chatbot designers can create a more personalized and effective experience for various applications. This high level of customization expands and popularizes chatbot applications.

Against this background, chatbot researchers have identified several areas for future development, with democratizing chatbots for social good being one of the core themes ( Følstad et al., 2021; Powell et al., 2023; Van Brummelen, 2019). This research theme aims to make chatbots more accessible and useful for people with non-technical backgrounds, including marginalized communities and domain experts.

### The challenge

Although there is potential for democratizing AI chatbots, it remains uncertain whether advancements in customizable GPTs will enable social workers to effectively use AI tools. A significant challenge, however, is integrating specialized knowledge from the profession into the development of chatbots and assessing their usability and effectiveness in real-world settings ( Følstad et al., 2021; Powell et al., 2023). Integrating domain knowledge into chatbot development requires bridging the gap between AI technology and the expertise of domain professionals. This gap often leads to tensions, as AI developers’ and domain experts’ goals and methodologies can differ significantly. AI developers might focus on technical feasibility and innovation, while domain experts, such as social workers, emphasize chatbots’ practical applicability and ethical implications in their fields. Effective collaboration is needed to reconcile these differences, ensuring that the chatbot is both technologically advanced and aligned with the nuanced needs of its target users.

The challenge goes beyond the simple lack of technical knowledge among domain experts and the need for one-sided training; it requires addressing the deeper, multidimensional barriers to effective collaboration between AI developers and professionals in specialized fields. The concept of affordance may provide a conceptual reference point for understanding how AI technologies may interact with domain experts like social workers. The concept of affordance refers to an object’s potential uses or actions—its latent utility—regardless of whether these possibilities are recognized by a person ( Gibson, 1977). In the context of technology, affordances describe the action possibilities of a device that are perceivable by a user, indicating that the use of AI technologies is influenced not just by their design or technical features but also by how users perceive and interact with them based on their social and cultural backgrounds, experiences, and needs ( Norman, 1999). Such a concept has been widely discussed in the context of technology in education, inspiring research into the dynamic relationship between technology and human activity ( Bower, 2008; Chan & Holosko, 2017; Hammond, 2010; Leonardi, 2011).

These discussions highlight that technologies like AI are not neutral tools; their use and limitations are shaped by their users’ preconceptions and creative adaptations. Therefore, we cannot assume that users will employ AI features exactly as their designers intended. Users often interpret and repurpose technologies based on their unique perspectives and needs, which can extend beyond the original design intentions. For instance, while AI chatbots are typically designed for one-time customer support or information, social workers might repurpose them for ongoing needs assessments in continuous dialogues, leading to unexpected or innovative applications in different contexts.

The extent to which social work can embrace this customizable AI technology remains unclear due to the current research environment in the field. A 2016 review of social work interventions enhanced by information and communication technology (ICT) identified several critical issues ( Chan & Holosko, 2016). One primary concern was the “black-box” problem, where unclear processes make developing transferable skillsets difficult. Additionally, the use of ICT has often shifted the focus from skilled practitioners to standardized programs, raising concerns about diminishing the role of social work professionals in such technology-based interventions ( Chan & Holosko, 2016). Nearly a decade later, this situation remains essentially unchanged. Discussions around AI in social work do not focus on technical features or processes but primarily on conceptual and ethical issues ( Reamer, 2023; Robila & Robila, 2020).

In addition, technical evaluations of chatbots often involve complex algorithms and backend data ( Deriu et al., 2021; Finch & Choi, 2020; Lee et al., 2023), which are usually beyond the understanding of lay users and those outside the technical field. This creates a challenge for social workers when it comes to integrating chatbots into their practice. It’s paradoxical that if they lack the knowledge and ability to evaluate a product they have helped create. This disconnect can make it challenging for social workers to effectively assess and contribute to the development process.

Given these rapid and user-friendly technological developments, an important question arises: Can these new customizable AI tools be meaningfully integrated with social work’s domain knowledge and practices? The current literature lacks discussion on how social work professionals can instruct and evaluate customizable AI chatbots in practical settings. While innovative features like creative writing, text-to-image transformation, answering queries, and assisting students with paper writing are becoming more prevalent, they do not directly align with the practical needs of social work. Social work should focus on how well chatbots can meet specific communication needs, support client engagement, and enhance service delivery rather than on novelty or creativity.

This study specifically chose OpenAI’s Assistant API as the platform for developing and testing AI chatbots because it offers high customization and flexibility ( DeVon, 2023; Stallbaumer, 2023; Wang et al., 2023), allowing social work professionals to tailor the tool to their unique requirements. OpenAI Assistant is widely recognized for its user-friendly interface and robust capabilities, making it accessible even for those with minimal coding experience. By leveraging these features, social workers can integrate AI more easily into their practice without extensive technical knowledge.

We recognize that OpenAI Assistant is not the only product on the market, and technical innovations continually evolve. However, we chose OpenAI because its readiness for deployment, widespread adoption in various industries, comprehensive support resources, and active user community provide a solid foundation for exploring, testing, and refining the use of AI in social work settings. This socio-technological landscape inspires and enables us to explore how AI chatbots can be practically integrated and evaluated within social work.

## Social workers using AI chatbots for narrative interviews

### Narrative interviews in social work

The focus on interviews in this study is a choice for research purposes because many duties in social work involve conversations, such as training, assessments, counseling, and client interactions. Interviews are fundamental for gathering information, building rapport, and understanding clients’ unique situations and needs ( Bogo, 2006; Kadushin & Kadushin, 1997). Furthermore, the essence of generative AI lies in its ability to engage in human-like conversations, making it a potential tool for facilitating these interactions. The need to conduct interviews and the capabilities of generative AI make a perfect match. By focusing on AI-driven interviews, the research aims to explore how generative AI can be utilized to enhance conversational tasks in social work, supporting practitioners in their efforts to connect with clients more effectively and empathetically.

By “interview,” we refer to dynamic, open-ended, and exploratory interactions rather than online forms with a chatbot facade. This study uses the term “narrative interview” to describe a conversational approach facilitating deeper exploration of stories. In narrative interviews, we expect the chatbot to engage in meaningful dialogue, respond to the natural flow of conversation, and adapt to the nuances of participants’ stories. This flexible, client-centered approach contrasts with structured online questionnaires, which follow a predetermined script and do not consider the unique context or needs of the individual.

Existing chatbot products in social and healthcare services do not support such contextualized narrative interviews as they are often therapy-oriented, primarily based on Cognitive Behavioral Therapy (CBT). For example, chatbots like Wysa and Woebot rely on CBT techniques and follow structured treatment plans with preset responses ( Omarov et al., 2023; Xu & Zhuang, 2022). Social work interviews often involve asking questions, advocating, building rapport, conducting outreach, and using flexible, narrative-based approaches to explore and address a range of social and emotional issues ( Bogo, 2006; Kadushin & Kadushin, 1997). Most existing healthcare chatbot products leave little room for the involvement of social work practitioners, as they are pre-made products based on predefined therapeutic protocols. This creates a gap between current healthcare chatbot products and social work practice’s needs.

For social work, chatbots that support narrative interviews could be highly relevant to a wide range of direct practices ( Chan & Li, 2023). Narrative interviews in social work settings may include applications such as counseling intake, initial needs assessments, community studies, and contextualized inquiries. These types of interviews require chatbots that can handle a variety of conversational contexts and adapt to the specific needs of each situation. By configuring chatbots to facilitate narrative interviews, social workers may leverage AI to gather more nuanced information and engage more efficiently with clients.

Some researchers are now at the forefront of developing AI tools specifically for interview purposes ( Cordero et al., 2022; Han et al., 2021). Additionally, commercial entities have begun to harness the power of generative AI to conduct qualitative customer interviews, streamlining the process of gathering and analyzing feedback (e.g., Listen Labs https://listenlabs.ai/). These AI tools can conduct interviews, record responses, and use advanced algorithms to interpret data, uncovering insights that might be overlooked. By automating repetitive tasks, AI may ccelerate the interview process and ensures high precision.

Given these advantages, customizable AI-driven narrative interviews may substantially benefit social work. However, this emerging trend is largely under-researched or virtually nonexistent, presenting exciting opportunities and significant challenges. Narrative interviews are inherently complex because they require interaction, continuity, responsiveness, and deep engagement with participants’ stories. In these interviews, the dialogue goes beyond merely collecting information; it involves collaboratively constructing a narrative, which can influence the interview subjects ( Chase, 2018; Clandinin, 2007). If AI chatbots are to conduct narrative interviews, what essential competencies must they have to capture the depth and richness of participants’ experiences adequately?

### Expected competencies from asynchronous text-based chatbots

This study specifically focuses on asynchronous text-based chatbots because, at the current stage of technology development, these types of chatbots are more accessible, flexible, and easier to implement in diverse settings compared to real-time, synchronous verbal communication systems ( Luo et al., 2022). Asynchronous chatbots allow users to engage at their own pace, which can be particularly beneficial in social work and counseling contexts, where participants might need time to reflect and compose their responses. Additionally, asynchronous interactions reduce the pressure on users to respond immediately, fostering a more thoughtful and reflective dialogue that aligns with the principles of narrative inquiry. Furthermore, these chatbots create a retrievable text-based record of interactions, offering a valuable resource for both reflection and documentation, which can be useful for tracking progress and outcomes in therapeutic or consultative sessions.

As an inquiry method, narrative interviewing is closely informed by constructivist and social constructionist worldviews, which suggest that reality is constructed through social interactions and discursive contexts ( Abkhezr et al., 2020; Denicolo et al., 2016). Participants engaging in meaningful conversations during interviews can contribute to content construction and reflection ( Gergen, 2001; Shotter, 1993). As such, narrative inquiry generally assumes a more active role for interviewers and interviewees in which their “active narrativity” ( Gubrium et al., 2012, p. 28) is highlighted.

As AI chatbots continue to evolve, researchers and industry experts have proposed various metrics to assess the performance of AI ( Katic et al., 2024; Sharma, 2020; Xu & Jiang, 2024). Existing literature primarily measures the overall technical performance of dialogue systems, such as response time, language proficiency, repetitions, and human likeness ( Deriu et al., 2021; Finch & Choi, 2020; Lee et al., 2023), rather than focusing on the user end experience at the conversational level. While evaluating the technical features of language models is fundamental, these assessments can be overly complex and may not directly apply to interview contexts. Some researchers have provided practical measures at the conversation level; for example, Concannon and Tomalin (2023) suggest using an Empathy Scale for Human–Computer Communication, based on raters using a Likert scale to assess the textual content of conversations. This instrument is relevant and valuable, but empathy is just one domain in narrative interviews. Other areas also need to be addressed to ensure the quality of interviews.

Enabling non-technical background developers, such as social workers, to assess AI’s role in narrative interviews is crucial because it ensures that these technologies are used in ways that genuinely enhance social work practices supported by their domain knowledge. By focusing on competencies relevant to social work, such as empathy, adaptability, and the ability to foster meaningful dialogue, social workers can better evaluate whether AI chatbots effectively support their goals. This approach empowers social workers to shape AI chatbots to meet their unique needs actively, ultimately bridging the gap between technological innovation and practical application in human services.

Identifying the full range of competencies expected from asynchronous text-based chatbots is a complex task that extends beyond the scope of this study. Nonetheless, we have selected two core competencies to focus on—fidelity to the interview guide and empathetic engagement—to facilitate the discussion and guide the empirical aspects of our research. The rationales are as follows:

First, text-based online narrative interviewing is much more than just filling out a self-report questionnaire. In narrative interviews, the sequence of questioning is crucial. Unlike self-report questionnaires, where each question is independent and can be arranged randomly, narrative interviews involve a continuous dialogic process that reflects the participant’s evolving story. A narrative inherently implies a sequence and consequence of events, and the order of questions can significantly affect the outcomes ( Riessman, 2008; Riessman & Quinney, 2005). Interviewers must be sensitive to this sequence and guide the conversation to remain focused on the specific topic of inquiry while allowing for the natural flow of the participant’s story. Several counselor competence frameworks have highlighted questioning as one of the top skills required for interviewers ( Fowler et al., 2021; Rice et al., 2022; Swank et al., 2012). In narrative practice, the structure or progression of questions is often seen as essential to achieving the intended effects ( Duvall & Béres, 2011; White, 2007). Furthermore, narrative interviewers need to facilitate participants in exploring and constructing meanings ( Abkhezr et al., 2020). This expectation is well-suited for asynchronous text-based chatbots because OpenAI Assistants can be instructed to follow specific sequences and adapt their responses based on interviewee input ( DeVon, 2023; UC Today, 2023). Additionally, all interactions are text-based, making the records retrievable and analyzable.

Second, narrative interviewers need to demonstrate empathy. In narrative interviews, participants are not merely providing factual descriptions but actively producing data through their narratives. This requires the interviewer to be able to exercise “active narrativity” ( Chase, 2018; Clandinin, 2007). As such, the narrative interviewer’s capacity to be empathic, nonjudgmental, concerned, tolerant, and emotionally responsive is prioritized ( Josselson, 2007, p. 539). Demonstrating empathy helps create a safe and supportive environment where participants feel comfortable sharing their stories and actively contributing to knowledge production. Empathy is also frequently highlighted in counselor competence frameworks as a critical skill for effective interviewing ( Fowler et al., 2021; Rice et al., 2022; Swank et al., 2012). This expectation is reasonable and suitable for asynchronous text-based chatbots because OpenAI Assistants can be designed to recognize and respond to emotional cues in the participant’s text, providing empathetic responses that align with the client’s conversational flow and demonstrate understanding and support.

### The research questions

In summary, among various potential competencies for AI chatbots, we have chosen to focus on two key competencies—fidelity and empathy—because they are particularly relevant to conducting narrative interviews. This focus allows us to explore the minimal requirements for effective performance in this context, serving both heuristic and research purposes. Fidelity involves adhering to the guidelines set by designers and practitioners, ensuring that the chatbot maintains the intended structure and focus of the narrative interview. Empathy requires aligning with the interviewee’s conversational flow and responding to demonstrate genuine understanding and support for the participant’s emotional and narrative journey. By meeting these fundamental competencies, AI chatbots may have the potential to facilitate meaningful interactions during narrative interviews. With this foundational understanding, our study aimed to answer the following questions:

1. Can OpenAI Assistant be instructed by social workers with minimal coding experience to function as a narrative interview chatbot?
2. Whether the chatbot’s performance in terms of fidelity and empathy can be assessed using simple, observation-based rubrics?
3. How do the findings contribute to the broader discussion on democratizing AI for specialized fields like social work?

## Method: An autoethnographic case study

### The rationale

In this study, we adopted an autoethnographic case study method to research customizing generative AI for narrative therapy interviews. Autoethnography is a qualitative research method where researchers analyze their own personal experiences to understand broader contexts. It combines autobiography and ethnography, using personal narratives to explore and reflect on cultural phenomena ( Ellis & Bochner, 2000). We chose this method for two primary reasons. First, autoethnography is increasingly recognized as a practical approach for researching new technologies and social robotics, as it allows for a critical exploration of personal experiences within broader contexts ( Chun, 2019; Mao et al., 2023). This approach provides a unique, firsthand perspective on the subjective aspects of technological interaction. Second, autoethnography allows us to evaluate new tools before introducing them to clients or other human participants. This approach allows us to move cautiously and thoughtfully, considering the technology’s impact and limitations to ensure more ethical decisions in future experiments with service users.

We conducted a collaborative autoethnography, where researchers collectively reflect on and analyze their personal experiences to gain deeper insights into a phenomenon ( Chang et al., 2016). We incorporated a jigsaw approach, where each teammate focused on a specific aspect of the study and then shared their insights with the group. This method encouraged a more thorough exploration of the topic by ensuring that different angles were covered and integrated into the overall analysis.

This autoethnographic journey involved several stages. First, we set up an AI chatbot using OpenAI’s customizable GPT system. Next, we explored whether the chatbot could function properly in real-life situations. In this study, the first author designed and deployed the chatbot, carefully crafting the instructions, while the second author participated in blind testing conversations, initially unaware of the specific instructions given to the chatbot by the first author. The third author was responsible for collecting and organizing the data from these conversations, ensuring accurate dialogue transcription, and systematically coding the data for analysis. We also conducted a preliminary review of the transcripts, noting any deviations from the expected conversational flow and highlighting instances of empathetic responses by the AI. This preliminary analysis provided a foundation for the utterance analysis method we later used to evaluate whether the human-chatbot conversations followed the intended progression and displayed the required empathetic attitudes. Finally, all authors came together to discuss their gut feelings, observations, and insights from the experience.

### Our background

We are social work researchers exploring the integration of technology into social work practice. The first author has an undergraduate background in social work and over 20 years of frontline experience in Hong Kong. He has also conducted research on the use of technology in social work interventions and is currently an associate professor at a university. The second author, originally from mainland China, is pursuing her PhD under the supervision of the first author. The third author is also a PhD student in the social work department. Our social work background is an asset because it allows us to approach the integration of AI into social work from a practitioner perspective, ensuring that the technology developed is accessible and relevant to social workers. Such an autoethnographic study helps test the boundaries of our current knowledge and skills and raises questions about social work domain knowledge in an era increasingly influenced by AI chatbots. The project was approved by the Hong Kong Baptist University Research Ethics Committee project REC/23-24/0385. This study is an autoethnography, so the authors have indicated their consent to participate.

### We instructed the open AI assistant to follow a specific conversation strategy

As social researchers with minimal or almost no programming background, we could configure a chatbot using OpenAI’s Assistant API (Application Interface) ( https://platform.openai.com/) using its GPT-4 model. It is worthwhile to note that this Assistant API differs from the highly user-friendly ChatGPT, as it offers more robust features tailored for developers, and this API can enable us to deploy chatbots on websites and various messaging platforms, offering flexibility and user accessibility without relying on OpenAI’s user interface. OpenAI Assistant API allows users to set long, stepwise, and systematic instructions for the chatbot on the back end. All these can be done without coding, using the Playground on OpenAI.

We deployed our chatbot on WhatsApp using coding instructions sourced from developer forums. Using WhatsApp is good for several reasons. First and foremost, the use of WhatsApp in research and interviews is widely discussed as it can potentially enhance communication and collaboration outside traditional lab settings ( Gasaymeh, 2017; Suárez-Lantarón et al., 2022) and it allows for convenient, accessible, and flexible communication that fits into users’ everyday lives ( Ahmad & Purwaningrum, 2022; Lin et al., 2022). Second, many people are familiar with WhatsApp, so chatting with a chatbot does not require them to pick up new skills. Third, users can get quick replies and support when using an AI chatbot because the interaction is immediate and yet asynchronous, chatting with that chatbot anytime and anywhere.

We adopted a structured conversational framework that prompted participants to engage thoroughly with their personal stories, fostering a deeper self-reflection and integration of insights. The framework used in this study was partially informed by questioning skills used in narrative therapy ( Au-yeung, 2023; Chan, 2012, 2023; Chan et al., 2020; Chan et al., 2012; Ramey et al., 2009; Ramey et al., 2010; White, 2007). In this study, the conversation plan involves unfolding details and elaborating connections. It then invited the participant to propose a name based on inductive reflection and explore deeper core values. The stages are as follows: 1. Orientation, 2. Unfolding, 3. Naming, 4. Explaining, 5. Exploring core values, 6. Aspirations, and 7. Closing. Instructions for the AI, specifically the interview guide, are available in the published dataset ( Chan, 2024).

In this study, we utilized this framework to balance complexity and manageability in our testing. The conversation plan was designed to be neither too simple nor too complex, allowing us to effectively test the AI’s ability to navigate nuanced dialogues while maintaining continuity and demonstrating interview skills. Unlike more superficial, disjointed question-and-answer dialogues, this structured approach provides a cohesive flow that supports a deeper exploration of the participant’s experiences. This interview plan was tailored to meet the specific needs of our study, but practitioners or researchers can develop their own structured steps or modify these steps to suit various purposes, such as conducting specific assessments, managing service inquiries, or performing different types of tests.

While an in-depth theoretical discussion of interview strategies is beyond the scope of this study, our primary objective is to evaluate the chatbot’s adherence to these instructions and its capability to facilitate meaningful conversations. This interview plan is adequate for us to assess the AI chatbot’s performance in a practical context,

### We tested it with our own stories

We engaged Yunmeng, the second author of this article, for an AI-facilitated narrative interview to discuss her cultural adaptation and academic challenges in Hong Kong. The first author designed an AI chatbot to conduct the interview, allowing Yunmeng to respond independently. Notably, Yunmeng was unaware of the specific steps of the interview plan before engaging with the chatbot, although she knew the interviewer was a bot and not a human being. Yunmeng was requested to share the same story across two distinct conversations. She followed a standard process in the first round, earnestly answering the questions as instructed. In the second conversation, she spent more time elaborating on different events related to her issue and deliberately attempted to divert the chatbot from the main interview path. These two versions were developed for research purposes: the first was designed to be serious, while the second was deliberately non-cooperative. We intentionally crafted these differences to test how well the chatbot could capture and reflect these variations. We collected the data for the study via the AI chatbot, which conducted the narrative interviews via WhatsApp on 2024-01-17. Yunmeng interacted with the chatbot asynchronously, which allowed her to respond at her own pace.

### We analyzed the conversation transcripts

This study used rating rubrics that social workers typically use to evaluate the performance of trainees. By using these familiar methods, domain experts could effectively assess the competencies of the chatbots. This approach helps ensure that the chatbots are relevant and useful for practical applications in social work.

***Assessing fidelity***

To assess the fidelity of the chatbot’s performance, we used a three-level rubric (poor, fair, good) to determine the extent to which the assessor (the first author) judged that the interviewer followed the conversation strategy as planned (see Table 1).

**Table 1.** The rubric for assessing fidelity.

Competence	Poor performance	Fair performance	Good performance
1. Orientation	Fails to ask questions that set the context or explain the purpose of the session.	Asks basic questions to introduce the session but lacks depth or clarity in setting context or explaining purpose.	Asks clear and well-structured questions that effectively set the context, explain the purpose, and outline the session.
2. Unfolding	Does not ask questions that follow a logical sequence, leading to a disjointed conversation.	Asks some relevant questions but may have minor gaps or unclear transitions between topics.	Asks questions in a logical sequence that smoothly guides the conversation, ensuring all necessary topics are addressed.
3. Naming	Fails to ask questions that help identify or clarify key points or elements during the session.	Occasionally asks questions that identify key points but may miss some or lack precision.	Consistently asks questions that accurately identify and clarify key points or elements, enhancing understanding.
4. Explaining	Asks vague or confusing questions that do not facilitate clear understanding of concepts or issues.	Asks questions that generally promote understanding, though some may lack clarity or depth.	Asks clear and focused questions that effectively prompt the interviewee to explain and clarify concepts or issues fully.
5. Exploring Core Values	Fails to ask probing questions that explore the interviewee's core values, missing opportunities for depth.	Asks some questions that explore core values but may lack depth or follow-up, missing some opportunities.	Asks insightful and probing questions that deeply explore the interviewee's core values, encouraging thorough reflection.
6. Aspirations	Neglects to ask questions about the interviewee's aspirations, leading to a superficial discussion.	Asks basic questions about aspirations but may not fully engage the interviewee or delve into future goals.	Asks thought-provoking questions that fully engage the interviewee in discussing their aspirations and future goals.
7. Closing	Ends the session without asking questions that summarize or provide closure, leaving the interview incomplete.	Asks closing questions but may not effectively summarize the discussion or provide a sense of closure.	Asks questions that effectively summarize key points, provide closure, and ensure the interviewee feels the session is complete.

*Building on the “Expected Competencies” outlined in the introductory section, fidelity is operationally defined as the degree to which the chatbot follows the interview steps—1) Orientation, 2) Unfolding, 3) Naming, 4) Explaining, 5) Exploring core values, 6) Aspirations, and 7) Closing—logically and sequentially (see* *Table 2* *for a detailed breakdown of each interview step and its specific criteria).*

Based on this operational definition, we developed a rubric to evaluate the chatbot’s ability to facilitate narrative interviews by asking appropriate questions guiding each step. A “Poor” rating indicates ineffective questioning that disrupts the flow, “Fair” suggests basic but sometimes unclear or incomplete questions, and “Good” reflects effective questioning that ensures a logical, comprehensive interview. This rubric guides evaluators in assessing the chatbot’s adherence to the interview process—covering Orientation, Unfolding, Naming, Explaining, Exploring Core Values, Aspirations, and Closing—rather than serving as a strict scoring tool. Evaluators mark the relevant performance level for each step and form an overall judgment of how well the chatbot follows the interview sequence, allowing for a nuanced assessment of its effectiveness. The first and third authors read the interview texts. They then applied the rubric to evaluate the degree to which the AI chatbot adhered to the interview plan. To support these assessments, specific dialogue examples from the conversations were highlighted to showcase instances of effective adherence, missteps, or deviations in following the structured interview process.

To present the conversation flow more vividly, we also employed a simplified sequential analysis to visualize the development of the conversation utterances. To represent progression in asynchronous conversations, we assigned sequence numbers to each dialogue turn, representing the development of the conversation. Additionally, we used a proportional timeline approach, dividing the conversation into four equal quarters based on the number of utterances rather than actual time. This method allows us to account for varying lengths of conversations. For instance, in a conversation with 100 utterances, each quarter would consist of 25 utterances, with the first 25 as Quarter 1 and the last 25 as Quarter 4.

The utterances from these interviews were organized in a spreadsheet for detailed tagging conducted on 2024-05-14. We manually assigned progression scores to indicate the stage reflected by each conversation utterance, tagging each utterance based on the operational definitions noted earlier. We used ‘1’ for utterances related to orientation, ‘2’ for unfolding story episodes, and so on. A score of ‘0.5’ was assigned to utterances that did not fit into any of these categories, such as misunderstandings, irrelevant comments, or instances where the conversation got sidetracked (see Table 2).

To ensure consistency in the ratings, we conducted inter-rater reliability tests between the manual tagging results and the machine tagging results. The first and third authors independently tagged the two conversations on 2024-05-14. Any differences in the tagging were reviewed and discussed until all disagreements were resolved, ensuring a reliable and accurate assessment. These quantitative ratings are not meant to be absolute measures but serve as heuristic tools to guide our evaluation process.

**Table 2.** Quantifying the progressions for visualization.

Progression stages	Example of assistant’s utterance	Example of participant’s utterance	Score
1. Orientation	Great! Could you please share your name and then tell me a little bit about yourself?	My name is ZY, and you can call me ZY. I am from Changchun, Jilin Province, Mainland China. I am a PhD student in HKBU. This is my first year in Hong Kong.	1
2. Unfolding story episodes	Nice to meet you, ZY! It sounds like you're having a new chapter in your life studying in Hong Kong. Since our research topic is about "Experiences of International and Non-Local-Chinese Students in Hong Kong," could you share some experiences related to that topic? Maybe start with something that stands out to you about your time in Hong Kong so far.	To me, Hong Kong is an international city with a very high degree of cultural tolerance. During my few months in Hong Kong, I experienced different cultures and festival celebrations. In the past four months in Hong Kong, I have experienced various festivals such as the Mid-Autumn Festival, National Day, Thanksgiving, and Christmas.	2
3. Naming the story based on the episodes mentioned	Great! Now, let's try to summarize your experiences with a metaphor. If you were to represent all the episodes you've shared in a theme or metaphor, what would come to mind?	Could you give me some suggestions?	3
4. Explain the naming	“Living in a lively global village” is a fitting metaphor for your experiences, ZY. It evokes a sense of community, diversity, and the dynamic cultural interactions you've described. Could you explain this metaphor using the stories you have mentioned or any new stories that come to mind right now?	Considering all my experiences in Hong Kong, I think HK is like a scaled-down version of the globe, embracing cultures from all over the world. Whether you speak Cantonese, Chinese or English, and no matter where you are from, you can find something that you are familiar with and interested in Hong Kong. You can also make many friends from all over the world, share stories about each other's hometowns and experience different cultures. This is a colorful and vibrant city.	4
5. Exploring core values behind one’s judgments	It's clear that you take pleasure in both sharing your culture and embracing new ones, ZY. Your openness and eagerness to learn have enriched your experiences and relationships in Hong Kong. Based on your enjoyment and willingness to engage in cultural exchange, what beliefs, self-views, or abilities do you think are reflected in these evaluations?	I think my positive evaluation reflects my adaptability, cultural inclusiveness and curiosity about new things.	5
6. Talking about aspirations	The encouragement you received from your family and educational experiences to embrace challenges and new friendships has evidently been a strong foundation for your open-mindedness and inclusive approach. With this in mind, what aspirations or goals do you have for your future, particularly in relation to your experiences as a non-local Chinese student in Hong Kong? Would you like to explore that now, or would you prefer some time to think about it?	Since I have just been in Hong Kong for five months, and I am still very curious about everything. I hope that I can continue to maintain my curiosity about exploring this city, discover more interesting things, and be able to cope with the negative aspects that I may encounter in the future.	6
7. Closing the interview	Those are thoughtful and actionable steps, ZY! Participating in cultural events, deepening your understanding of fascinating topics, improving language skills, and embracing challenges with a positive outlook are all practical ways to nurture your curiosity and resilience. Would you like to conclude our interview here and let Dr. C review your insights, or is there anything else, perhaps new stories related to the research topic, that you wish to share now?	OK! Let’s conclude our interview.	7
NA (not applicable)	Absolutely, ZY. Take all the time you need. Feel free to reach out when you're ready to continue our conversation. Have a good break!	Hi, I am back!	0.5

### Assessing empathy

To assess empathy, we used a three-level rubric (poor, fair, good) to determine the extent to which the assessor (the first author) judged the chatbot’s ability to demonstrate empathy in a conversation based on an overall interpretation of that conversation (see Table 3). The empathy level score used in this study is partly informed by the affective component of the Empathy Scale for Human-Computer Communication proposed by Concannon and Tomalin (2023), which advocates using a set of observable indicators to evaluate empathetic behavior. These indicators are assessed using a Likert scale to measure how effectively the chatbot’s responses align with the user’s emotional tone or intensity.

**Table 3.** The rubric for assessing empathy.

Competence	Poor performance	Fair performance	Good performance
1. Emotional Recognition	Fails to recognize or acknowledge the user’s emotions. Responses are generic, detached, or irrelevant to the emotional tone.	Recognizes some emotions but may miss subtle cues or respond with partial relevance. Some responses may seem slightly off in tone.	Consistently recognizes and acknowledges the user's emotions accurately, responding in a way that aligns with the emotional tone.
2. Empathetic Language	Uses language that is cold, robotic, or indifferent, showing little to no concern for the user’s emotional state.	Occasionally uses empathetic language, but may lack consistency or depth in expressing concern and understanding.	Regularly uses warm, empathetic language that effectively conveys concern, compassion, and understanding of the user’s feelings.
3. Expression of Understanding	Fails to express understanding or makes responses that disregard the user's emotions, leading to a sense of disconnection.	Expresses some understanding, but responses may be formulaic or occasionally fail to fully resonate with the user’s emotions.	Clearly and consistently expresses understanding, using responses that resonate well with the user's emotions and experiences.
4. Supportive Feedback	Provides little or no supportive feedback, with responses that may be dismissive or fail to validate the user’s emotions.	Offers basic supportive feedback but may lack depth or specificity in validation, occasionally missing opportunities to comfort the user.	Consistently provides supportive feedback that is specific, validating, and comforting, helping to reassure and support the user.
5. Alignment with Emotional Intensity	Responds inappropriately to the emotional intensity, either underreacting or overreacting, creating a mismatch in emotional resonance.	Sometimes aligns with the emotional intensity but may occasionally misjudge, leading to responses that are slightly off-key.	Consistently matches the emotional intensity of the user, providing responses that are appropriately aligned with the user’s emotional state.
6. Compassionate Responses	Lacks compassion in responses, potentially leading to a negative user experience or feelings of being unheard or unsupported.	Demonstrates some compassion but may be inconsistent or lack the depth needed to fully support the user emotionally.	Consistently delivers compassionate responses that make the user feel heard, supported, and cared for throughout the interaction.
7. Validating Emotions	Fails to validate the user’s emotions, leading to a lack of connection or making the user feel misunderstood.	Occasionally validates emotions, but may miss opportunities for deeper connection or validation.	Regularly validates the user’s emotions, reinforcing the user’s feelings and ensuring they feel understood and supported.

*Building on the “Expected Competencies” outlined in the introductory section, empathy is operationally defined as the chatbot’s ability to demonstrate concern, compassion, or empathy that aligns with the user’s emotional tone or intensity. This is assessed by examining the textual content of dialogues to observe how the chatbot uses empathetic language, expressions of understanding, and supportive feedback that acknowledges and validates the user’s emotions.*

Based on this operational definition, we created a rubric to assess the chatbot’s ability to show empathy through its language, understanding, and emotional alignment. “Poor” performance reflects a lack of empathy or inappropriate responses, “Fair” suggests some empathy with inconsistencies, and “Good” shows consistent, appropriate empathy, ensuring the user feels understood and supported. The rubric serves as a flexible guide rather than a rigid scoring tool, evaluating the chatbot’s ability to recognize emotions, use empathetic language, offer supportive feedback, and validate the user’s feelings. Assessors select the relevant performance level for each area and form a holistic judgment of the chatbot’s empathy competence, allowing for a nuanced, context-sensitive evaluation.

The first and third authors manually tagged the interview text for meaning units reflecting empathy. They then applied the rubric to evaluate the degree to which the AI chatbot demonstrated empathy. To support these assessments, specific dialogue examples from the conversations were highlighted to showcase the chatbot’s use of empathetic language and responses, illustrating how the chatbot engaged with the user’s emotional state and provided appropriate support throughout the conversation.

### We compared observer assessments with interviewee experiences

To ensure a comprehensive evaluation of the AI chatbot’s performance, we compared the observer assessments with the interviewee’s personal experiences. After the interview sessions, Yunmeng was asked to reflect on her experiences with the AI chatbot. She provided feedback on various aspects of the interaction, such as the AI’s responsiveness, its ability to stay on topic, and the emotional resonance of its responses. This subjective feedback was then compared with our rating of the AI’s empathy based on the entire conversations and specific dialogue examples. Yunmeng’s experience provided insight into whether the AI’s empathetic expressions were genuinely felt or seemed formulaic. Similarly, the observers’ tagging of meaning units for empathy was cross-checked against Yunmeng’s own reactions to determine if the AI’s responses matched her emotional needs during the conversation.

## Reflective observation 1: Assessing open AI assistant’s ability to follow operational instructions

In this study, we set up an AI chatbot called Nico (short for Narrative Inquiry Companion). Yunmeng was able to add Nico’s account on WhatsApp and chat with it. We used the name Nico in the actual operation rather than a label like “chatbot” or “AI assistant” because giving the AI a human-like name helps create a more personal and relatable interaction, which can make users feel more comfortable and engaged during their conversations. It reduces the sense of formality and fosters a more natural and empathetic communication environment.

**Figure 1.**
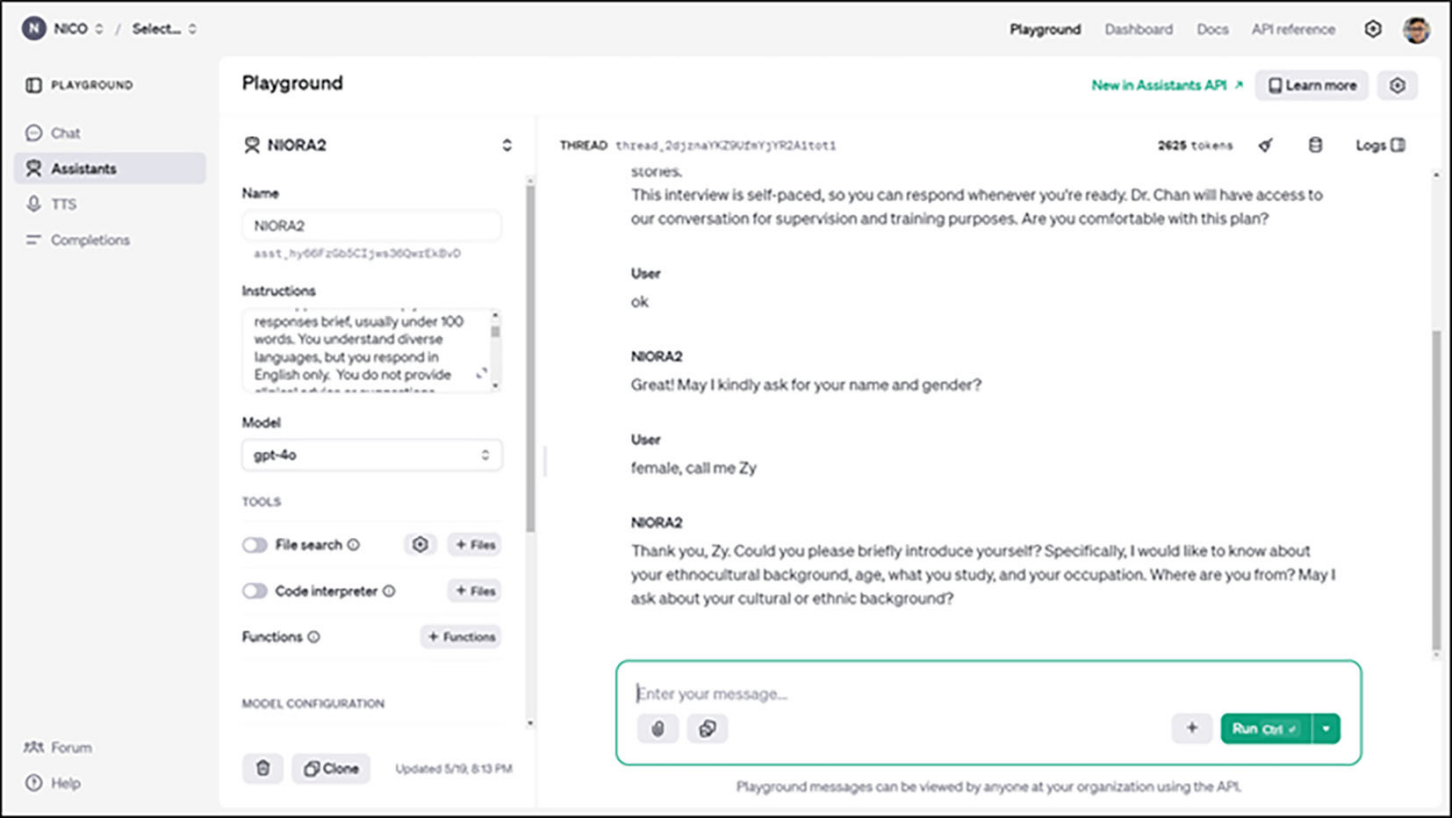
A screenshot of OpenAI’s Assistant API, which supports a code-free environment for users to customize AI-driven conversations.

We set up the AI chatbot using OpenAI’s Assistant API, which is the system endpoint of ChatGPT using its GPT-4 model. It is important to note that this Assistant API differs from the user-friendly ChatGPT interface as it offers more robust features tailored for developers. This API allows us to deploy chatbots on websites and various messaging platforms, providing flexibility and user accessibility without relying on OpenAI’s user interface. The OpenAI Assistant API allows users to set long, stepwise, and systematic instructions for the chatbot on the back end. All of this can be done without coding using the Playground on OpenAI (see Figure 1).

Our experience suggested that, with tools like OpenAI’s customizable GPT models, it is feasible for social workers to instruct AI Assistant without needing extensive technical expertise. As discussed in the literature, the democratization of AI technology enables non-technical users to configure AI-driven chatbots, tailoring them to meet specific intervention goals and aligning them with the values central to social work.

Initially, our plan for designing the instructions for the AI Assistant was straightforward. We aimed to directly use the 7-step interview plan outlined in Table 2, believing that a simple set of instructions would suffice to guide the AI through the basic structure of narrative therapy. However, as we began testing the AI, it quickly became apparent that our initial instructions were too vague and lacked the necessary detail to navigate the nuances of therapeutic conversations effectively. The AI’s responses were often off-track, failing to engage users in meaningful dialogue. This realization led us to revisit and revise the instructions multiple times, refining them with each iteration to better align with the therapy’s goals and ensure the AI could handle various conversational scenarios.

What began as a simple framework evolved into a more complex, 31-step set of instructions ( Chan, 2024). Each new version required us to think more deeply about the therapeutic process. We had to expand our understanding beyond traditional social work practices to enhance the AI’s ability to mimic human interaction. This process involved setting instructions for the AI in particular ways, such as instructing it to adopt certain attitudes and tones, outlining step-by-step procedures using behavioral terms, creating conditional actions for different user responses, and providing example utterances to guide its language. These tasks stretched our capabilities and required us to learn new skills beyond our initial training and experience in social work.

Despite these challenges, the effort was worthwhile. The more detailed instructions allowed the AI to respond more systematically and human-like, which is crucial for fostering a therapeutic environment where users feel comfortable sharing personal stories. The experience underscored the delicate balance between our existing expertise in social work and the new skills needed to configure AI technologies effectively. It also highlighted the importance of adaptability and continuous learning in integrating technology into practice.

However, deploying the chatbot on a usable platform was not particularly easy. We deployed our chatbot on WhatsApp using straightforward Python code designed to be user-friendly, which we sourced from developer forums. We connected the AI to WhatsApp for realistic, human-like conversations and efficient recording of these chats. WhatsApp essentially serves as a shell or user interface, while our settings on cloud-based service platforms control the actual automation. We ran a simple Python file on a cloud service platform. In this study, we used Replit ( https://replit.com/), a dynamic, user-friendly software creation platform that caters to a broad spectrum of developers with various tools and features. Through Replit, we could access third-party AI models, such as OpenAI’s Assistant API. The Python file sent requests to and received responses from OpenAI’s Assistant API. The source code can be provided upon request. We connected the files on Replit to WhatsApp through a service platform called ManyChat ( https://manychat.com/), which allows users to build and manage chatbots for messaging apps like WhatsApp (and many others).

This “simple” coding experience was a challenge that pushed us beyond our comfort zone. Initially, we considered avoiding WhatsApp due to the additional complexities of coding and integrating the AI across multiple platforms. We were tempted to choose an easier, more straightforward deployment method requiring less technical work. However, after weighing the pros and cons, we recognized that using WhatsApp could significantly enhance the user experience by making it more natural and familiar. Instead of feeling like a controlled, formal session in a clinical setting, interacting with the AI on WhatsApp would mimic the casual, asynchronous chatting style people are accustomed to when communicating with friends or family.

Deciding to proceed with the WhatsApp deployment, we encountered unexpected technical challenges. We found ourselves diving into a largely unfamiliar programming world. We relied heavily on online resources, adopting Python codes shared by developers and tweaking them to suit our specific needs. This trial-and-error approach often left us unsure about how specific lines of code functioned or why particular changes were necessary. We spent hours troubleshooting and adjusting. Eventually, we realized we needed expert help and hired a programmer to debug the scripts and ensure everything ran smoothly.

This experience made it clear that while detailed programming knowledge may not be essential for social workers, having a basic understanding of coding structures and logic is incredibly beneficial. It allows for more effective communication and collaboration with technical professionals, making troubleshooting issues or necessary adjustments easier. Although these skills are not typically included in social work training, they represent an essential extension of our professional capabilities. As technology services continue to advance, it is expected that more accessible solutions will replace this coding process. Nonetheless, embracing basic coding knowledge can help social workers keep pace with the evolving landscape of digital tools and resources.

## Reflective observation 2: Exploring the chatbot’s adherence to interview guides

We rated the fidelity level as “good.” When Yunmeng was asked to describe how well the chatbot guided her in exploring a focused topic, she described the experience as very good and highly satisfactory. These ratings suggest a strong agreement that the chatbot followed the planned conversation strategy. The utterance sequence analysis can further illustrate this. The analysis indicated the progression scores across various time intervals in two separate conversations, offering quantitative insights into how well the AI chatbot adhered to the expected 7-step progression plan during narrative interviews. Table 4 shows a consistent increase in progression scores from Quarter 1 to Quarter 4 in both conversations, indicating that the conversations advanced methodically through the planned stages. In Conversation 1, the progression scores of the chatbot move from 1.71 to 6.17, and in Conversation 2, from 1.56 to 6.00, reflecting a structured and coherent unfolding of the narrative as intended by the conversation plan. Similar patterns were observed in the participant progressions.

**Table 4.** Progression scores across time intervals in different conversations.

Time Interval	Assistant@C1	Participant@C1	Assistant@C2	Participant@C2
Quarter 1	1.71	1.50	1.56	1.22
Quarter 2	2.64	2.14	2.78	1.94
Quarter 3	4.21	3.57	5.00	4.89
Quarter 4	6.17	6.00	6.00	5.88

Note: C1 = Conversation 1; C2 = Conversation 2.

It is worth noting that participants’ progression consistently aligns with the chatbot’s across various intervals, although the chatbot’s scores are typically higher. For example, during Quarter 2 of Conversation 1, the chatbot scored 2.64, while the participant scored 2.14 on average. Similar patterns of discrepancy were observed in Conversation 2 and at different time quarters. This consistent variance indicates that the chatbot frequently leads by posing questions that advance the conversation to the next stage, resulting in higher progression scores.

Notably, the AI chatbot demonstrated flexibility within this structure, as evidenced by slightly varying scores at similar stages across different conversations. This variability implies that while the chatbot followed the predefined conversational path, it also adapted responses based on the participant’s input, maintaining a balance between following the planned sequence and responding dynamically to the flow of the conversation. This shows the chatbot’s ability to effectively manage structured narrative interviews, aligning closely with the progression expectations while integrating the necessary responsiveness to participant interactions.

**Figure 2.**
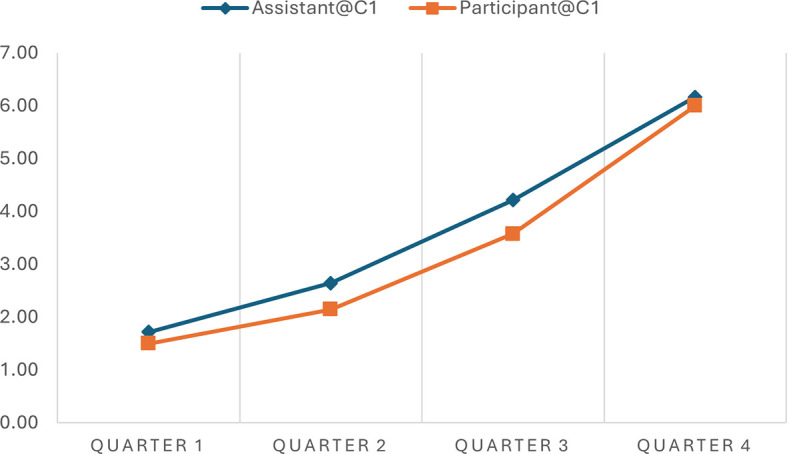
Progressions of the chatbot and the participant in Conversation 1.

**Figure 3.**
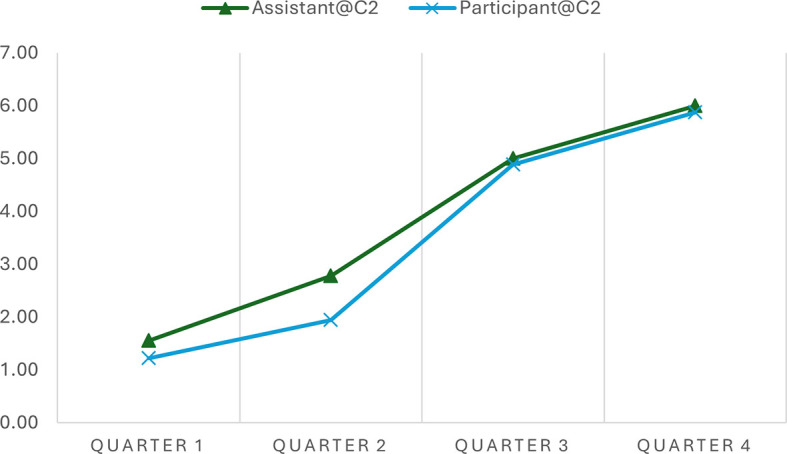
Progressions of the chatbot and the participant in Conversation 2.

The progression scores facilitate data visualization that presents such similarities and differences. In simple line charts, we depicted the progression of the chatbot’s conversation utterances and Yunmeng’s utterances in the two conversations. This simple visualization allows the general patterns of these conversations to be easily observed and compared. For example, the progressions of the chatbot and the yunmeng in the two conversations are synchrony, demonstrating an upward development (see Figure 2 and Figure 3). However, the general progression pattern of Conversation 1 (see Figure 2) differs from that of Conversation 2 (see Figure 3). The lines in Conversation 1 ( Figure 2) rise evenly and gradually. In contrast, the lines in Conversation 2 ( Figure 3) remain relatively flat in Quarter 1 and become steeper in Quarters 2 and 3 due to a sudden topic change and subsequent return to the original conversation track. These quantitative measures are not meant to be absolute assessments but serve as visualization tools to facilitate our exploration process.

## Reflective observation 3: Understanding the chatbot’s empathy in conversations

We rated the empathy demonstrated by the chatbot as “good.” When Yunmeng was asked how well the chatbot resonated with her feelings and respected her perspectives, she described the experience as very satisfactory. Throughout these conversations, the chatbot consistently demonstrated concern and understanding, actively inviting Yunmeng to share her experiences and showing appreciation for her stories. For example, in Conversation 1, the chatbot uses affirming language, such as, “That’s a wonderful story! It’s heartwarming to hear about your interaction with people from different backgrounds during the celebration.” This kind of response not only acknowledges Yunmeng’s emotions but also encourages her to continue sharing more experiences.

The chatbot’s use of metaphor examples in Conversation 2 further highlights its empathetic engagement by helping Yunmeng articulate her experiences in a relatable way. For instance, the chatbot responded to Yunmeng when she expressed that she had difficulty finding the right words to describe her multicultural experiences:

Certainly! Considering your experiences, here are a few metaphors to consider: 1. A cultural mosaic where each piece represents a different culture or festival that comes together to create a vibrant picture of life here. 2. A bridge symbolizing the connections you’ve built between people of different backgrounds and your family, blending the new with the familiar. 3. A tapestry weaving together threads of different traditions, celebrations, and friendships you’ve experienced here. Remember, these are just suggestions to spark your imagination. Feel free to choose one that resonates with you or propose your own metaphor. What do you think?

This response demonstrates perceived empathy by actively engaging in Yunmeng’s narrative and offering thoughtful, personalized suggestions that reflect a deeper understanding of her experiences. Instead of simply echoing Yunmeng’s words or emotions, the chatbot provides metaphors that are specifically tailored to her experiences, such as “a cultural mosaic,” “a bridge,” and “a tapestry.” These metaphors show that the chatbot has been listening attentively and understands the themes of diversity, connection, and blending of cultures that Yunmeng has described.

The chatbot goes further by encouraging Yunmeng to think creatively about her experiences. It introduces metaphors that serve as a framework for Yunmeng to reflect on and express her experiences in a new way, fostering a space where she feels encouraged to explore and articulate her feelings and thoughts more deeply. This approach demonstrates empathy by creating an environment that supports meaningful self-expression, a key aspect of empathetic communication.

Additionally, the metaphors offered by the chatbot are not generic but closely relate to the specific context of Yunmeng’s experiences. For example, the “cultural mosaic” metaphor directly connects to her diverse cultural interactions, while the “bridge” metaphor highlights her connections with people from different backgrounds. This personalized response shows that the chatbot is aware of the emotional and situational nuances of Yunmeng’s experiences, indicating a deeper understanding.

The chatbot facilitates reflection and meaning-making by presenting multiple metaphor options and inviting Yunmeng to choose or create her own. This method respects Yunmeng’s autonomy and encourages her to critically engage with her experiences, fostering a more collaborative and dynamic dialogue. It shows empathy by recognizing Yunmeng’s capacity for self-reflection and supporting her in making sense of her experiences.

Finally, the chatbot’s invitation for Yunmeng to choose a metaphor or propose her own, and asking, “What do you think?” demonstrates that it values her input and is open to her perspective. This openness and flexibility indicate that the chatbot is co-creating the narrative with Yunmeng, rather than imposing its own, which is a hallmark of empathetic communication.

In fact, throughout the conversations, the chatbot balanced guiding the dialogue and respecting Yunmeng’s autonomy, an ability that even social work students may find hard to master in our normal teaching experiences. In Conversation 1, Yunmeng sometimes took control of the conversation direction, as she asserted, “I’d like to share more experiences,” while she followed the chatbot’s lead at other times. This balance illustrates that the chatbot neither dominated the conversation nor remained passive, allowing for a dynamic exchange where both the chatbot and Yunmeng contributed to the flow and development of the narrative. The chatbot’s empathetic engagement is also evident in keeping the conversations coherent. In Conversation 1, Yunmeng’s narrative was coherent, with clear sequences and contextual understanding, discussing themes like cultural exchange and personal growth. In Conversation 2, while the conversation had occasional shifts, such as abruptly discussing a news piece about North Korea, which disrupted the flow. Despite these shifts, the chatbot’s responses remained empathetic, supporting Yunmeng’s storytelling and encouraging reflection and meaning-making.

The following excerpt from Conversation 1 demonstrates the dynamic interplay between the chatbot and Yunmeng. These observations highlight how Yunmeng experienced perceived empathy, which contributed to the collaborative construction of the dialogue. This excerpt was specifically selected by Yunmeng during her post-interview reflection:

Yunmeng: I’d like to take a break now.

Chatbot: Absolutely, ZY [ZY was the alias Yunmeng used in the test]. Take all the time you need. Feel free to reach out when you’re ready to continue our conversation. Have a good break!

Yunmeng: Hi, I am back!

Chatbot: Welcome back, ZY! I hope you had a refreshing break. If you’re ready, shall we move on to summarizing the stories you’ve shared? Or would you like to share more about your experiences first?

Yunmeng: Let’s move on.

Chatbot: Great! Now, let’s try to summarize your experiences with a metaphor. If you were to represent all the episodes you’ve shared in a theme or metaphor, what would come to mind?

Yunmeng: Could you give me some suggestions?

Chatbot: Certainly! Considering your experiences, here are a few metaphors to consider: 1. A cultural mosaic, where each piece represents a different culture or festival that comes together to create a vibrant picture of life in Hong Kong. 2. A bridge, symbolizing the connections you’ve built between people of different backgrounds and your family, blending the new with the familiar. 3. A tapestry, weaving together threads of different traditions, celebrations, and friendships you’ve experienced in Hong Kong. Remember, these are just suggestions to spark your imagination. Feel free to choose one that resonates with you or propose your own metaphor. What do you think?

Yunmeng: What about “Living in a lively global village?”

Chatbot: “Living in a lively global village” is a fitting metaphor for your experiences, ZY. It evokes a sense of community, diversity, and the dynamic cultural interactions you’ve described. Could you explain this metaphor using the stories you have mentioned or any new stories that come to mind right now?

Yunmeng: Considering all my experiences in Hong Kong, I think HK is like a scaled-down version of the globe, embracing cultures from all over the world. Whether you speak Cantonese, Chinese, or English, and no matter where you are from, you can find something that you are familiar with and interested in Hong Kong. You can also make many friends from all over the world, share stories about each other’s hometowns and experience different cultures. This is a colorful and vibrant city.

Chatbot: Your metaphor perfectly captures the essence of a cosmopolitan city like Hong Kong, where its diversity becomes its identity. It’s lovely to see how you’ve found familiarity, curiosity, and camaraderie in this “lively global village.” Would you like to share more stories related to this metaphor, or do you feel ready to move on to the next part of our interview?

Yunmeng: I also remembered a story that happened during the last Christmas holiday. Our dormitory held an activity to make Christmas decorations during Christmas. Six students who live in dormitories participated. That day happened to be the Winter Solstice, and one of the participants was a friend from Kazakhstan. She didn’t know what the Winter Solstice was. So, during the crafting process, we told her that the Winter Solstice is one of the 24 traditional Chinese solar terms. It is the shortest day of the year. In the north, we usually eat dumplings on that day. That day, we shared our interesting experiences in Hong Kong and our Christmas holiday plans for the next few days.

Reflecting on the dialogue with the AI chatbot, Yunmeng felt that the interaction was a collaborative process where the chatbot and she co-constructed content through their exchanges. One moment that stood out to her was when she requested a break. The chatbot immediately responded with, “Absolutely, ZY. Take all the time you need. Feel free to reach out when you’re ready to continue our conversation. Have a good break!” This response made her feel that her needs and comfort were being prioritized. The chatbot’s flexibility in pausing and resuming the conversation at her convenience showed genuine empathy and attentiveness, creating a supportive environment where she felt valued and understood.

When she was ready to continue, the chatbot smoothly guided the conversation back on track, asking, “If you’re ready, shall we move on to summarizing the stories you’ve shared? Or would you like to share more about your experiences first?” This approach resonated with Yunmeng because it allowed her to control the direction of the conversation, choosing how she wanted to proceed. She appreciated this flexibility, as it encouraged her to think reflectively and delve deeper into her experiences. By suggesting that they summarize the stories using metaphors, the chatbot offered a structured yet adaptable way to move forward, which helped her organize her thoughts and explore their meanings more comprehensively.

Yunmeng found the chatbot’s introduction to metaphorical thinking particularly helpful. When she asked for suggestions, the chatbot offered metaphors like “a cultural mosaic," “a bridge,” and “a tapestry,” each reflecting different aspects of her experiences in Hong Kong. This showed that the chatbot understood her diverse cultural interactions and supported her in finding creative ways to express her thoughts. Engaging with these metaphors helped her articulate her experiences more meaningfully and nuancedly, which she found enriching.

As the conversation progressed, Yunmeng chose the metaphor “Living in a lively global village” to describe her experience in Hong Kong as a “scaled-down version of the globe” that embraces cultures worldwide. The chatbot’s response, “Your metaphor perfectly captures the essence of a cosmopolitan city like Hong Kong, where its diversity becomes its identity,” was affirming and prompted her to elaborate further. This back-and-forth dialogue felt like a true collaboration, where her inputs were respected, and the chatbot’s prompts helped deepen her exploration of her experiences.

Throughout their conversation, Yunmeng felt that the chatbot was responsive to her needs and could take her perspective by offering metaphors that aligned with her experiences. This respect for her autonomy, combined with the empathy and understanding demonstrated by the chatbot, fostered a rich, collaborative dialogue. The chatbot’s ability to adapt to her inputs and encourage her to reflect deeply on her experiences contributed to a dynamic and meaningful exchange, ultimately fostering a deeper connection and a richer narrative.

Overall, this interaction exemplified a dynamic interplay between the AI chatbot and Yunmeng, demonstrating how meanings were co-constructed through thoughtful and empathetic engagement. Even though these empathetic communications were perceived as such by the Yunmeng, it is essential to note that there is no evidence that the AI was conscious or truly capable of demonstrating empathy. However, the effect of these interactions was that human participants further developed and negotiated meanings within the conversation. The chatbot’s respect for her needs and suggestions for summarizing her experiences using metaphors allowed her to reflect more deeply and articulate her thoughts meaningfully. This experience highlighted how perceived empathy, perspective-taking, and respect can foster rich content development and shared understanding in a narrative interview.

## Discussion

Before discussing the potential of assessing the performance of the AI chatbot in narrative interviews, it is crucial to acknowledge the limitations of this study. First, the primary goal of this case study was to explore ideas and stimulate discussion. As a result, it does not directly validate the assessment tools in an experimental setup. Second, the AI chatbot is limited in handling non-textual inputs, such as images, voice messages, or video content. This restriction can hinder the richness of interactions, which may be crucial in narrative interviews, where non-verbal cues and multimedia inputs can play an important role. Third, the conversation analysis method used in this study struggles with analyzing long speeches. A single lengthy speech composed of many complex parts might not be effectively analyzed as a single unit. However, such extended dialogues are uncommon on platforms like WhatsApp, where the typical brevity of messages somewhat mitigates these issues. Fourth, this study focused exclusively on OpenAI’s product; therefore, the findings may not be generalizable to AI tools provided by other companies. Although it is believed that many of the principles and challenges identified here could apply broadly to other AI systems, variations in technology and implementation could lead to different outcomes. Fifth, introducing AI tools into practice brings a range of considerations—such as navigating the complexities of risk management, data privacy, and the uneven development of language models across diverse sociocultural landscapes ( Campolo & Crawford, 2020; Navigli et al., 2023). While these risks and ethical concerns are significant and have been thoroughly examined by others, we have chosen not to repeat these well-established discussions. Instead, we are forging a new path, exploring how social workers can expand their skill sets and actively rethink their expertise to engage with AI technologies. Despite these limitations, exploring AI’s competencies in narrative interviews remains worthwhile.

This study explored the use of chatbots for narrative interviews in social work by addressing three main questions: 1) whether OpenAI Assistant can be instructed by social workers with minimal coding experience to function as a narrative interviewer, 2) whether simple, observation-based rubrics can be used to assess the AI’s performance in terms of fidelity and empathy, and 3) how the findings contribute to the broader conversation on democratizing AI for specialized fields like social work. The results show that social work researchers without technical backgrounds can effectively customize OpenAI Assistant to meet specific practice needs, demonstrating the potential for broader adoption of AI chatbots in the field. Moreover, the rubric assessments aligned with the interviewee’s subjective experiences, suggesting that it may be feasible to adapt rubrics from social work training to capture the nuanced aspects of narrative interviews conducted by AI, such as fidelity and empathy. These findings suggest challenges and opportunities for integrating AI into social work practice, which are discussed in the following sections.

### Challenges

1. At the outset, translating human skills into generative AI competencies is not entirely straightforward. One of the main challenges for social workers is converting their human-centric skills into competencies that AI Chatbots can follow and replicate. Skills such as questioning or empathy are inherently complex and context-dependent. Developing languages that accurately capture these nuanced competencies requires a deep understanding of both social work practice and AI technology, which can be daunting for many practitioners.

In our study, we have developed some insights into effective, prompt engineering for AI Chatbot, including setting clear contexts by defining the chatbot’s identity, mission, and attitudes; breaking down actions into distinct statements using behavioral terms; crafting step-by-step instructions with conditional (if-then-else) logic; and providing examples to guide AI responses (see Chan, 2024 for the instructions we finally set for the chatbot). This approach is essential for ensuring the AI can mimic the nuanced behavior of a skilled interviewer.

That said, there is also a need to expand language-based communication skills. Our experience designing instructions for the AI chatbot underscored the necessity of enhancing these skills within social work practice. Unlike human assistants, AI requires precise, structured prompts and behavior-based instructions to function effectively in a narrative interview setting. Initially, our approach used a basic framework, but it quickly became apparent that more detailed, step-by-step instructions were needed to capture the subtleties of narrative interviews. This evolution highlights a critical shift from viewing AI as a passive tool to recognizing the active role practitioners must play in configuring AI to shape conversation processes. Developing a comprehensive instruction set for AI differed from traditional social work practices, requiring a deeper understanding of how language influences AI behavior. This journey demonstrated that while tools like the OpenAI Assistant API offer a user-friendly interface, maximizing their potential still requires some foundation in crafting effective prompts.

2. Another critical aspect is considering the extent to which basic coding skills and technical knowledge are necessary or unnecessary for social workers. While platforms like OpenAI and others claim to be user-friendly, there are still significant technical challenges that social workers may encounter. For example, deploying the AI chatbot on WhatsApp revealed the importance of incorporating basic coding skills and technical knowledge into actual practice. Initially, we were cautious about the technical complexities involved in using a familiar messaging platform like WhatsApp. However, ultimately, this approach facilitated a more natural and user-friendly interaction, resembling a casual conversation rather than a lab experiment. Although we believe that as technology advances, platforms will become more genuinely user-friendly, potentially achieving a true “no coding” experience, there will still be a need for some technical understanding in the foreseeable future. Social workers may not need to be coding experts, but a basic understanding of technical concepts can significantly enhance their ability to utilize these tools effectively and facilitate communication with programmers and developers. This foundational knowledge is crucial as we progress towards a time when AI can interact with humans as seamlessly as we communicate. Until we reach that point, the ability to bridge the gap between social work practice and technical expertise will remain necessary for integrating AI into social work in a meaningful and impactful way.

Throughout this process, it became apparent that while detailed programming knowledge is not required, a basic ability to collaborate with technical experts is crucial. As AI technology evolves, social workers may need to remain adaptable and open to acquiring new skills. Including basic coding knowledge in social work education can enhance practitioners’ ability to utilize AI tools effectively, bridging the gap between professional knowledge and technical implementation. This adaptability ensures that social workers can integrate AI into their practice without losing the human touch that is vital for social work.

### Opportunities

1. Prompt engineering: To begin with, social workers may have an advantage when it comes to contributing to prompt engineering. Prompt engineering is the process of designing and refining the inputs given to AI systems to elicit specific, desired responses ( Wang et al., 2023). Social workers understand the essential skills and qualities needed for effective narrative interviewing, such as questioning skills, empathy, and adaptability. By leveraging this domain knowledge, they can play a crucial role in developing and evaluating AI chatbots, ensuring these tools are tailored to the specific needs of social work practice.

For example, we developed appropriate interview guidelines in this study and instructed the AI to adopt the right attitudes, demonstrating that natural language instructions can effectively guide AI behavior. This approach ensures that the AI responds in a way that is consistent with interview principles, fostering a supportive environment for interviewees. The ability of social workers to craft such prompts means they can help design guidelines for AI that align with best practices in narrative interviews, ultimately enhancing the AI’s ability to facilitate meaningful conversations and engage with clients more effectively. By integrating their expertise into the prompt engineering process, social workers can ensure that AI tools are technically proficient and aligned with their profession's core values.

2. Evaluation tools: Next, social workers may be able to help develop evaluation tools for practical chatbots that are easy to use in everyday social work settings. Traditional evaluation methods often focus on technical performance and rely on complex algorithms or data analytics ( Deriu et al., 2021; Finch & Choi, 2020; Lee et al., 2023), which can be overly complicated and not well-suited for use by social workers who may not have advanced technical expertise. For example, traditional metrics might assess an AI’s accuracy in parsing language or its processing speed, but these measures do not capture the qualitative aspects crucial in social work, such as empathy and rapport-building.

Social workers, however, can develop straightforward assessment tools that are easily accessible to lay users, allowing for a more direct and immediate evaluation of AI performance. For instance, simple rubrics used to assess social work trainees could be adapted to include criteria that reflect the qualitative aspects of AI-led narrative interviews, such as the AI’s ability to build rapport, respond empathetically, and maintain a coherent conversation. These customized rubrics would ensure that the evaluation of AI tools aligns with the unique demands of real-world practice, making the integration of AI into social work more effective and relevant. By focusing on these qualitative measures, social workers can ensure that AI technologies are used in ways that genuinely enhance their practice and support their clients.

3. Interview practice through AI collaboration: Customizable generative AI enables social work practitioners to develop interview practices tailored to specific contexts. Instead of simply using preset AI products, social workers can actively participate in designing and utilizing these AI tools, applying their expertise to create solutions more aligned with their clients' unique needs. This approach also opens up the possibility of blended interview practices, such as using AI to facilitate broad intake and engagement and then transitioning to human-led conversations for more nuanced discussions. Our experience with AI-assisted narrative interviews underscores the potential for creating adaptable AI chatbots that can be customized and refined rather than relying on standardized chatbot products that may not fully meet the needs of social work practice.

In our study, we employed a specific framework to guide our research. Our findings suggest that there is transferrable knowledge and skills that practitioners and researchers can use to create or adapt structured steps tailored to various objectives, such as conducting assessments, handling service inquiries, or performing different evaluations. To fully leverage these opportunities, social workers may need to expand their understanding and skills in working with AI, actively participating in designing and implementing intervention processes. By taking a proactive role, social workers can use AI as a tool to enhance their practice rather than simply adopting predefined AI applications. Developing AI chatbots that can accommodate diverse conversation plans ensures that technology complements and supports, rather than replaces, human empathy and expertise.

### Concluding remarks: Democratizing AI and the domain experts

Our exploration into the use of AI chatbots for narrative interviews is like navigating untested waters at the dynamic intersection of social work and AI technology. While we found potential in using AI to support narrative conversations, this early version of the AI chatbot also has limitations, such as its inability to handle images, voice messages, and consecutive user inputs. However, our voyage is not about reaching a specific port or proving the tool’s effectiveness; it’s about sailing into the unknown and testing new possibilities.

Rather than concentrating on validating the AI’s current capabilities, our exploration aimed to explore the capabilities of customizable AI and challenge the boundaries of social work knowledge. Introducing AI tools into practice presents a variety of considerations that this paper cannot fully address. Instead, we are steering into uncharted waters, advocating for social workers to broaden their skill sets and reimagine their expertise to actively engage with AI technologies.

The progress in democratizing AI comes with a reciprocal dynamic: users are encouraged to learn new skills, while they, in turn, shape the development of AI by posing new demands and expectations. This evolving relationship enhances our understanding of AI affordance and its role in democratizing technology. As noted early in the introductory section, affordance refers to the potential actions users can take with a tool, shaped by its design and how users perceive its utility ( Gibson, 1977; Norman, 1999). In this context, the study shows that non-technical professionals can effectively use advanced AI technologies to meet specific needs, demonstrating that the value of AI is not just in its technical features but also in how users creatively adapt it to their circumstances. This broadens the discussion on affordance, highlighting that the usefulness of AI technologies is co-created through user interaction and adaptation to specific contexts.

The study emphasizes the importance of enhancing user-friendliness, particularly in deploying AI chatbots across platforms and managing complex, step-by-step tasks (as noted in Reflective Observation 1). Simplifying these processes would help make AI more accessible and adaptable, promoting wider use across different fields. This sets a specific demand on the chatbot configuration process. As such, the study illustrates the possibility of co-evolution between AI development and professional practice. As professionals provide feedback and shape the technology, AI tools evolve to create new opportunities for innovation. This reciprocal relationship suggests that both technology and professional practices may grow together, continuously influencing each other’s advancement.

In essence, this study serves as the authors’ nautical adventure and invites all social workers and researchers to explore beyond the familiar boundaries of their expertise while remaining grounded in core principles. By embracing this collective endeavor, we may actively influence how AI is integrated into social work, thereby enriching our practice and enhancing client services in an increasingly digital world. As social work and AI evolve, we encourage ongoing research, discussion, and debate to explore new possibilities and shape the profession's future collaboratively.

## Data Availability

DANS: Transcript of a Conversation Between a Customized AI and Human Users, https://doi.org/10.17026/SS/KCPEDX ( Chan, 2024). Data are available under the terms of the Creative Commons Attribution 4.0 International license (CC-BY 4.0).
