# Supplementary material for: A Case Study on Assessing AI Assistant Competence in Narrative Interviews
Source: F1000Res. 2024 Oct 4;13:601. Originally published 2024 Jun 7. [Version 2] doi: 10.12688/f1000research.151952.2 (PMC11564924; doi:10.12688/f1000research.151952.2)
Supplement: Peer review history

# Reviewer report: Reviewer response for version 2

**DOI:** 10.5256/f1000research.171244.r334817

**Published:** 2025-01-02

**Authors:** Anna De Fina

**Affiliations:** 1 Georgetown University, Washington, DC, USA

I found the article overall very good both in presenting the justification for the research and the theoretical/methodological basis for the work. I also thought that the methodology and results were carefully explained and justified. The only changes that I would suggest are the following:

 1. I think the authors should better relate the  introductory part about the need to study the possible applications of AI developments to social work with the case of the interview. The interview case is presented as a separate issue after the introduction but I feel that these two parts of the article should be better weaved together.

 The authors should also devote a bit more space to explaining the model that they followed for designing a narrative interview and therefore also exemplify the different parts of the interview. They just name those parts (Naming, Orientation, etc.) but do not explain what they consist of.

 Finally, among the limitations of the study, they should add the fact that one of the authors was fully conscious of the background and aims of the experiment and might have been biased in their evaluation of the chatbox's performance.

Is the case presented with sufficient detail to be useful for teaching or other practitioners?

Yes

Is the work clearly and accurately presented and does it cite the current literature?

Yes

If applicable, is the statistical analysis and its interpretation appropriate?

Yes

Are all the source data underlying the results available to ensure full reproducibility?

Yes

Are the conclusions drawn adequately supported by the results?

Yes

Is the background of the case’s history and progression described in sufficient detail?

Yes

Reviewer Expertise:

Sociolinguistics, Discourse Analysis

I confirm that I have read this submission and believe that I have an appropriate level of expertise to confirm that it is of an acceptable scientific standard, however I have significant reservations, as outlined above.

# Reviewer report: Reviewer response for version 2

**DOI:** 10.5256/f1000research.171244.r329782

**Published:** 2024-11-14

**Authors:** Sunok Lee

**Affiliations:** 1 Aalto University, Espoo, Finland

This study employed a text-based interview with a single human participant using the WhatsApp messaging app, utilizing OpenAI’s Assistant API with the GPT-4 model. The AI assistant conducted two interview sessions: the first, where the participant was cooperative, and the second, where the participant attempted to sidetrack the AI. This approach aimed to assess the AI’s performance in narrative-based interviewing by evaluating its adherence to interview structure, empathy, and support for participant agency. Researchers used a set of predefined criteria informed by narrative interview literature to measure the AI's performance, including structural fidelity, empathy, and narrative coherence. The assessments were carried out by a human researcher and the AI, and inter-rater reliability was tested to validate consistency in the results.

**Strengths of the Study**

 The authors are to be commended for their innovative approach to studying AI’s potential in qualitative research, particularly in the complex area of narrative interviewing. Their setup of the AI assistant on WhatsApp, a familiar platform, reflects a practical and thoughtful choice, making AI accessible and potentially easier for social work practitioners to adopt. The decision to use predefined criteria from existing narrative interview literature for performance evaluation is well-grounded, allowing for a structured analysis that aligns with established qualitative research practices.

**Main Areas for Improvement**

1. **In-depth Ethical and Critical Issues Analysis**
  While the study provides a promising exploration of AI’s utility in conducting narrative interviews, it lacks a thorough discussion of potential risks, especially concerning interactions with vulnerable users. If AI is to be applied widely, especially in fields involving sensitive interactions, the paper must address the risks of incorrect or insensitive AI responses. The absence of this discussion leaves out critical considerations that might arise if AI were to replace human interaction, such as the potential harm caused by insensitive responses to vulnerable participants. A more comprehensive examination of these ethical issues, including possible measures to mitigate such risks, is essential for providing a balanced view.
2. **Interpretation of Progression Scores**
  Although progression scores were introduced to evaluate the interview’s structure, the authors’ interpretation could benefit from additional nuance. The current discussion seems overly definitive in concluding that the AI achieved meaningful progression in narrative structure. Given the limitations of a single participant case study, a more tentative interpretation would be appropriate. Furthermore, while progression scores offer a useful metric, the study could provide practical examples or case-specific explanations to clarify their real-world implications. This would give readers a clearer understanding of how these scores influence the AI’s interview performance and could enhance the study’s transparency.

**Other Areas for Improvement**

1. **Clearer Focus on Research Questions and Study Scope**
  The study attempts to evaluate both the AI assistant's performance and the validity of the assessment metrics, which causes some confusion. It would be beneficial to clarify the primary research objective and distinguish between assessing the AI’s performance and the reliability of the evaluation metrics. Additionally, the study would benefit from acknowledging its limitations upfront, particularly as a preliminary or pilot study, to set appropriate expectations for the reader.
2. **Balanced Perspective on AI's Potential in Narrative Interviews**
  The literature review highlights AI’s benefits in qualitative research but tends to overstate its potential, neglecting the ongoing debate around AI's limitations in qualitative settings. Acknowledging the contentious nature of AI in qualitative research would present a more balanced viewpoint. This would strengthen the paper by recognizing that while AI offers efficiencies, there are ethical and methodological concerns that still need resolution.

**Overall**

 This study offers valuable insights into AI’s capabilities in conducting narrative interviews, presenting an innovative approach to exploring AI applications in qualitative research. However, a more balanced perspective on the limitations, especially concerning ethical implications and interpretive caution, would enhance the study’s scientific rigor. Addressing these areas could improve the paper's structure and strengthen its value as a foundational study for further exploration in this field.

Is the case presented with sufficient detail to be useful for teaching or other practitioners?

Partly

Is the work clearly and accurately presented and does it cite the current literature?

Partly

If applicable, is the statistical analysis and its interpretation appropriate?

No

Are all the source data underlying the results available to ensure full reproducibility?

Yes

Are the conclusions drawn adequately supported by the results?

Yes

Is the background of the case’s history and progression described in sufficient detail?

Partly

Reviewer Expertise:

Human-centered AI, Participatory Design, Human-computer Interaction.

I confirm that I have read this submission and believe that I have an appropriate level of expertise to confirm that it is of an acceptable scientific standard, however I have significant reservations, as outlined above.

# Reviewer report: Reviewer response for version 2

**DOI:** 10.5256/f1000research.171244.r329218

**Published:** 2024-10-17

**Authors:** Neil Ballantyne

**Affiliations:** 1 Open Polytechnic of New Zealand, Lower Hutt, Wellington, New Zealand

Many thanks to the authors for investing the time to substantially revise and improve the article. I believe that all of the issues I raised, including the pilot status of the study, have now been addressed and the article is fit for indexing.

Is the case presented with sufficient detail to be useful for teaching or other practitioners?

Partly

Is the work clearly and accurately presented and does it cite the current literature?

Partly

If applicable, is the statistical analysis and its interpretation appropriate?

Partly

Are all the source data underlying the results available to ensure full reproducibility?

Yes

Are the conclusions drawn adequately supported by the results?

Partly

Is the background of the case’s history and progression described in sufficient detail?

No

Reviewer Expertise:

social work, human service technology, artificial intelliegence, qualitative research.

I confirm that I have read this submission and believe that I have an appropriate level of expertise to confirm that it is of an acceptable scientific standard.

# Reviewer report: Reviewer response for version 1

**DOI:** 10.5256/f1000research.166648.r298273

**Published:** 2024-08-15

**Authors:** Neil Ballantyne

**Affiliations:** 1 Open Polytechnic of New Zealand, Lower Hutt, Wellington, New Zealand

This single-subject case study examines the performance of an AI assistant conducting a narrative, text-based interview with a single human participant using the WhatsApp messaging app.

 The researchers designed the AI assistant using OpenAI’s Assistants API with the GPT-4 model. The interview was conducted twice under two different conditions. During the first, the human participant was instructed to cooperate with the AI assistant, and during the second, they were instructed to be disruptive, attempting to sidetrack the AI assistant in response to questions.

 To assess the performance of the AI assistant on the specific and complex task of narrative-based interviewing, the researchers designed criteria and measures that, based on a review of the narrative interview literature, they deemed to be relevant and valid measures of performance. They devised and applied measures addressing different dimensions of the narrative interview, including interview structure, empathy, narrative coherence, complexity, and support for the agency of the human participant.

 A human research assistant and ChatGPT 4 assessed the interview data using these measures, and the results were compared using an inter-rater reliability test. The principal researcher reviewed the results and resolved any disagreements.

**Strengths of the study**

 The researchers are to be commended for devising an imaginative research design to study a complex area of human-computer interaction in a subject area ­(the application of generative AI to qualitative interviewing) that is both highly topical and deeply controversial. The design of the AI assistant and its deployment on WhatsApp seems to have been well considered, and the limitations imposed by this short exchange, text-based platform are recognised. The criteria for selecting the key measures to assess narrative interview performance, drawn from the literature identified, makes sense. However, a whole paper could have been written on this aspect alone.

**Main areas for improvement**

***Be clear about research questions and primary focus***

 A lot is going on in this study, and the description and rationale for key design choices are sometimes unclear.  The problem begins with the abstract and the research questions, where there seems to be slippage between a focus on evaluating the performance of the AI assistant and evaluating the validity and inter-rater reliability of the measures constructed. The first research question asks:

*How can the proposed metrics help us, as social researchers without a technical background, understand the quality of the AI-driven interviews in this study?*

 In my view, RQ1 would be better focussed on assessing the validity and reliability of the measures selected for evaluating the performance of the AI assistant. This needs to be established before an actual performance assessment of the AI assistant is made. Establishing inter-rater reliability of the measures would be a part of that task.

 However, this raises the question of why the researchers decided to have only two raters, one human and the other a GenAI. The comparison is interesting but muddies the waters. This machine versus human rater aspect of the study was a surprise since it was not signalled in the abstract, introduction or research questions. A comparison between the ratings made by a GenAI and human raters would be far more interesting once we had established human inter-rater reliability, which could have been achieved with only a few more humans in the frame.

**Establish from the outset that this is a pilot study**

 The title and the abstract refer to the study as a case study, albeit one with a single participant, giving the reader the impression of a significant, in-depth exploration of a phenomenon. However, in the section on limitations, the authors describe the study as a pilot study and state that the primary goal “ *was to explore ideas and stimulate discussion rather than to conduct rigorous experiments”.*

 I recommend that the abstract and introduction be rewritten to reflect this stance. However, this does beg the question as to whether the paper might be better construed as a *working paper* for feedback and discussion than as a substantive academic article.  The paper is very provocative and stimulating in terms of the issues it raises but inconclusive as an assessment of the utility of an AI assistant for narrative interviews.

**Other areas for improvement**

***A  balanced approach to the literature and the claims made***

 The literature review is interesting and includes many relevant articles, but the articles selected and the authors’ comments are prone to over-accentuate the potential of a technology that is still emergent. The application of GenAI to qualitative research is deeply contested in the mainstream literature on qualitative methodology, making it hard to concur with the authors’ views that:

*The integration of generative AI has revolutionized and democratized the process of qualitative data collection, analysis, and interpretation. This technological leap has boosted efficiency and expanded the horizons of narrative inquiry methods.*

 The authors can improve the literature review by recognising GenAI's emergent nature and the fact that many scholars in the mainstream qualitative research field have strong reservations about its applicability and ethical concerns about its use.

***Other issues***

 There are several places in the text where the authors’ explanations of the methods used are a little too opaque. For example, the explanation about the progression scores was helpful but left me puzzled about how this was practically implemented. An example might help.

 Also, in the findings, the authors highlight that the participant exhibited higher agency during conversation two, which is surely explained by the instruction given to the participant to try to throw the interviewer off track. If that isn’t the explanation, a fuller account as to why not would be helpful.

**Overall**

 I want to encourage the authors to continue their exploration of the use of GenAI in qualitative research, and I believe that this is a very useful working paper that raises many pertinent issues. Responding to the comments above might help to improve the structure and flow of the paper, but the research design issues cannot be remedied without conducting a completely new study. It is for that reason I decided not to recommend the paper for indexing.

Is the case presented with sufficient detail to be useful for teaching or other practitioners?

Partly

Is the work clearly and accurately presented and does it cite the current literature?

Partly

If applicable, is the statistical analysis and its interpretation appropriate?

Partly

Are all the source data underlying the results available to ensure full reproducibility?

Yes

Are the conclusions drawn adequately supported by the results?

Partly

Is the background of the case’s history and progression described in sufficient detail?

No

Reviewer Expertise:

social work, human service technology, artificial intelliegence, qualitative research.

I confirm that I have read this submission and believe that I have an appropriate level of expertise to state that I do not consider it to be of an acceptable scientific standard, for reasons outlined above.

## References

[1] : A Case Study on Measuring AI Assistant Competence in Narrative Interviews. *F1000Research* .2024;13: 10.12688/f1000research.151952.1 10.12688/f1000research.151952.1 PMC1156492439555385

# Response

**Published:** 2024-09-07

**Authors:** Chitat CHAN

**Affiliations:** Social Work, Hong Kong Baptist University, Hong Kong, Hong Kong

**//Main areas for improvement**

***Be clear about research questions and primary focus***

**A lot is going on in this study, and the description and rationale for key design choices are sometimes unclear.  The problem begins with the abstract and the research questions, where there seems to be slippage between a focus on evaluating the performance of the AI assistant and evaluating the validity and inter-rater reliability of the measures constructed. The first research question asks:**

***How can the proposed metrics help us, as social researchers without a technical background, understand the quality of the AI-driven interviews in this study?***

**In my view, RQ1 would be better focussed on assessing the validity and reliability of the measures selected for evaluating the performance of the AI assistant. This needs to be established before an actual performance assessment of the AI assistant is made. Establishing inter-rater reliability of the measures would be a part of that task.**

**However, this raises the question of why the researchers decided to have only two raters, one human and the other a GenAI. The comparison is interesting but muddies the waters. This machine versus human rater aspect of the study was a surprise since it was not signalled in the abstract, introduction or research questions. A comparison between the ratings made by a GenAI and human raters would be far more interesting once we had established human inter-rater reliability, which could have been achieved with only a few more humans in the frame.//**

 *Response: The research questions and focus have been entirely reformulated. The revised manuscript now distinguishes its objectives. It focuses on the democratization of AI chatbots and the emerging trend of customizable AI, which has opened new opportunities across various fields, including social work. The study explores how social workers, particularly those with minimal coding experience, can instruct and evaluate AI assistants to conduct narrative interviews. The role of narrative interviews is used as a case to explore possibilities and provide feedback within a broader context. The emphasis is on qualitative assessment rather than quantitative measurement, aligning with the exploratory nature of the study.

**//Establish from the outset that this is a pilot study**

**The title and the abstract refer to the study as a case study, albeit one with a single participant, giving the reader the impression of a significant, in-depth exploration of a phenomenon. However, in the section on limitations, the authors describe the study as a pilot study and state that the primary goal “ *was to explore ideas and stimulate discussion rather than to conduct rigorous experiments”.***

**I recommend that the abstract and introduction be rewritten to reflect this stance. However, this does beg the question as to whether the paper might be better construed as a *working paper* for feedback and discussion than as a substantive academic article.  The paper is very provocative and stimulating in terms of the issues it raises but inconclusive as an assessment of the utility of an AI assistant for narrative interviews.//**

 *Response: The pilot and exploratory nature of the study is emphasized. The revised abstract and introduction explicitly state that the study is exploratory and serves as a pilot to stimulate discussion about the use of AI in social work. The manuscript now clearly outlines its objectives, focusing on the democratization of AI chatbots and the emerging trend of customizable AI. The study is qualitative and concerned with the extent to which domain knowledge experts can participate in instructing and assessing AI chatbots.

**//Other areas for improvement**

***A  balanced approach to the literature and the claims made***

**The literature review is interesting and includes many relevant articles, but the articles selected and the authors’ comments are prone to over-accentuate the potential of a technology that is still emergent. The application of GenAI to qualitative research is deeply contested in the mainstream literature on qualitative methodology, making it hard to concur with the authors’ views that:**

***The integration of generative AI has revolutionized and democratized the process of qualitative data collection, analysis, and interpretation. This technological leap has boosted efficiency and expanded the horizons of narrative inquiry methods.***

**The authors can improve the literature review by recognising GenAI's emergent nature and the fact that many scholars in the mainstream qualitative research field have strong reservations about its applicability and ethical concerns about its use.//**

 *Response: The literature review has been revised to acknowledge the democratization of AI chatbots and the emerging trend of customizable AI, which opens new opportunities across various fields, including social work. It addresses limitations alongside potential benefits. The discussion part addresses these limitations, noting that the study focused exclusively on OpenAI's product and that findings may not be generalizable to AI tools from other companies. While many principles and challenges identified here could apply broadly to other AI systems, variations in technology and implementation could lead to different outcomes. The introduction of AI tools into practice also brings considerations such as navigating risk management, data privacy, and the uneven development of language models across diverse sociocultural landscapes. While these challenges are important and have been extensively explored by others, the study focuses on exploring how social workers can expand their skill sets and rethink their expertise to engage actively with AI technologies.

***//Other issues***

**There are several places in the text where the authors’ explanations of the methods used are a little too opaque. For example, the explanation about the progression scores was helpful but left me puzzled about how this was practically implemented. An example might help.**

**Also, in the findings, the authors highlight that the participant exhibited higher agency during conversation two, which is surely explained by the instruction given to the participant to try to throw the interviewer off track. If that isn’t the explanation, a fuller account as to why not would be helpful.//**

 *Response: The revised manuscript provides clearer explanations of the methods used. It explains that the study is autoethnographic, reflecting personal experiences and using rubrics for assessing fidelity and empathy in the AI assistant’s performance. Examples of conversation transcripts are included to illustrate these qualitative assessments, emphasizing the depth and context of interactions rather than mere numerical data. We also enhanced clarity in findings. It was noted that some findings, like the participant exhibiting higher agency, were not well-explained.  In the revised discussion, the measure of agency has been removed and integrated into the discussion about empathy and a deeper exploration of meanings. This approach aligns with the study’s focus on qualitative assessment and provides a clearer understanding of the findings.

**//Overall**

**I want to encourage the authors to continue their exploration of the use of GenAI in qualitative research, and I believe that this is a very useful working paper that raises many pertinent issues. Responding to the comments above might help to improve the structure and flow of the paper, but the research design issues cannot be remedied without conducting a completely new study. It is for that reason I decided not to recommend the paper for indexing.//**

 *Response: We are very grateful for the thoughtful and constructive comments provided. We deeply appreciate the feedback, especially regarding the areas for improvement and the suggestion to frame the study as a pilot. These insights were particularly helpful in guiding us toward a more focused and balanced approach. We also want to acknowledge that the first version of the manuscript had several gaps, and we sincerely apologize for any confusion this may have caused. We recognize that the clarity and direction of the paper needed refinement, and we are thankful for the opportunity to improve. In response, we have rewritten the article using the same dataset but with a more refined focus, especially clarifying the research aims and scope. The revised study explores how AI chatbots, particularly GPT-4, can be applied in social work for conducting narrative interviews. Using an autoethnographic case study approach, social work researchers developed and tested an AI chatbot on WhatsApp. The chatbot followed a structured interview plan and demonstrated empathy, though challenges in replicating human skills within AI were noted. The study concludes that social workers can tailor AI tools for narrative interviews, but further research is needed to address the field's complexities. Our focus remains on democratizing AI and empowering social workers to use these tools effectively while emphasizing the need for interdisciplinary collaboration in developing robust assessment tools.

# Reviewer report: Reviewer response for version 1

**DOI:** 10.5256/f1000research.166648.r298274

**Published:** 2024-07-26

**Authors:** Jonathan Singer

**Affiliations:** 1 Loyola University Chicago, Chicago, Illinois, USA

This manuscript addresses an interesting and important topic. My comments are focused on clarifying certain details to enhance the paper’s clarity and coherence, rather than critiquing its main idea, which I find compelling but somewhat unclear in its current form. Despite reading through the entire paper multiple times, I struggled to fully grasp key parts. Reorganizing the literature review, introducing a clear “Methods” section, and streamlining the manuscript would greatly improve its readability.

**Literature Review**

 The literature review begins by emphasizing the potential of AI in narrative interviewing, but it would benefit from first defining and describing what narrative interviewing entails. The enthusiastic discussion of AI, including references to “influential bloggers,” feels more promotional than academic, especially since it turns out to be an advertisement for a for-profit company selling narrative interviewing tools. To strengthen the paper, I recommend moving the definitions of key terms like “narrative interviewing” and “conversation analysis” to the beginning of the literature review. Additionally, information from paragraph 5 and the section “Why do assistant’s interview skills matter” should be relocated to the start to provide important context early on.

 The language in the literature review occasionally seems exaggerated and inconsistent. For instance, the statement that AI is “becoming a crucial tool for researchers” feels overstated without supporting evidence. Furthermore, it appears contradictory to claim that AI improves analysis while also stating that academics have not “explored how AI could enable or limit narrative interviews.” Clarifying these points and providing supporting evidence will strengthen the argument.

 There are also some statements that seem unnecessary and detract from the narrative flow, such as “This brief review of narrative inquirer capabilities is not comprehensive.” Removing such remarks can streamline the text and improve readability.

**Methods Section**

 Introducing a “Methods” header before discussing competence metrics would improve the manuscript’s structure. The justification for the competence metrics needs to be clearer. Rather than stating that “The following metrics were chosen primarily for their simplicity and to facilitate testing and demonstration,” it would be helpful to explain the rationale behind selecting these specific metrics and whether they are based on existing frameworks or newly developed by the authors.

**Scores: **The authors identified six scores but did not talk about how those scores were established, the scales, or anything else about them. Specifically:

**Competence 1: **The explanation of how progression scores are assigned is vague. There is no clear description of the criteria used to determine the progression stage of each utterance. This lack of specificity makes it difficult to understand the validity and reliability of the scoring system.

 The method’s reliance on previous studies (Au-yeung, 2023; Chan et al., 2020; Connor et al., 2009; Moran et al., 2005; Ramey et al., 2009) without providing a detailed explanation or adaptation process is problematic. It would be helpful to have a more thorough, but not necessarily long explanation of how these methodologies are applied to the current study.

 Relatedly, the sequential analysis method from therapy research may not be directly applicable to narrative interviews conducted by AI. If this method was developed from narrative interviews, then say so. There are therapy approaches like CBT or behavioral therapies that have structured, goal-oriented sessions. ABFT is task oriented, but still provides the therapist structure. Narrative interviews, from your description, are more open-ended, possibly more like Rogerian or supportive therapy sessions. The adaptation of this method needs more rigorous justification.

 I was also unclear how and why a higher score isn’t better, and also why you calculated average progression scores over time. How does this score help us to understand how good AI is doing?

**Competence 2: **Is there empirical validation or psychometric testing of the empathy scale? If so, please provide that information for the reader. I would like more information about why a Likert scale is used for empathy, especially since the previous competence argued that higher scores were not necessarily better. That does not seem to be the case with this scale.

**Competence 3: **I didn’t understand the use of the word “Domination” in the subheader.

 You introduce multiple metrics (agency, coherence, complexity, and meaning-making) without clearly explaining how they are operationalized. Each one of these needs to be defined and the reader needs measurement criteria for each metric, especially since the previous two measures apparently used different scales. For example, the authors say that measuring “agency” cannot be done in isolation, but there is no description of how the scores or definitions of the other measures are used to understand or measure agency. Indeed, it seems that if the concepts in Competence 3 are interrelated, might there be a way to discuss the measures as a coherent package, rather than individual scales?

 The description of the Case study has many strengths, including the innovative use of ChatGPT to create a chatbot that the researchers would otherwise have not been able to program, highlighting the value of deploying the chatbot on WhatsApp, clearly defining the seven stages, and the ingenuity of having the postgraduate have two conversations – one genuine and one combative. The major limitation of the description of the case study is that it seems to be disconnected from the measures. I know that you provide results in the results section, but it would be helpful to have notes about how each section connects to the measures.

**Data Analysis Method and Data Collection Section**

 I was unclear about the context in which the postgrad was having the WhatsApp conversations. Was this in a lab? Were the researchers observing? Was this in private?

 I didn’t understand why the conversation was divided into four equal quarters. i.e. The choice of four quarters seems arbitrary. I know that it makes it easy to analyze by utterances rather than time, but it seems to miss the importance of space and silence in a narrative interview.

 The process for manually assigning progression scores is not clearly defined. There is no description of the criteria used by the human research assistant to assign these scores, which raises concerns about the subjectivity and consistency of the ratings. Is there any research on using LLMs or ChatGPT specifically to assigning scores to narrative interviews? If so, it would be good to cite that here.

**Results**

 The agreement between the human research assistant and ChatGPT4 on most metrics in and of itself is encouraging for researchers interested in using LLMs.

 In finding 2, what distinguishes a 3 from a 4? This is part of my ongoing concern about the paper’s lack of clarity with the measures.

 The disagreement on coherence in Conversation 2 highlights the need for clear criteria. The authors wrote, “In the rating, the human research assistant and ChatGPT4 mostly agreed on all these metrics, except that there was a disagreement on coherence in Conversation 2, where the human research assistant gave a score of 2 and ChatGPT4 gave a score of 3. After reviewing this, the principal researcher sided with ChatGPT4's score.” The manuscript should detail the specific aspects that led to different ratings and the rationale for siding with ChatGPT4’s score. Especially in these early days of using LLMs for analysis, this kind of transparency will be instructive as well as increase trustworthiness of the analysis.

**Discussion**

 I thought it was interesting that there were challenges with long speeches in conversation analysis. Even though WhatsApp tends to have shorter exchanges, what are some potential solutions or alternative methods to address these challenges, especially since narrative interviews often involve extended dialogue. I’m thinking specifically about qualitative researchers who are interested in using this for more traditional open-ended narrative interviews, not on WhatsApp. Perhaps this is part of an expanded discussion on the scalability of this type of AI use.

 I agree that there need to be more refined metrics (and some of my critiques of this paper are in line with that call).

 How would you propose to improve interrater reliability? Again, are there computational linguists who have looked into human/LLM IRR?

 After reading through the discussion a few times, I still didn’t have a good understanding of the differences between conversations 1 and 2. For example, in the results section you noted differences in agency, coherence and complexity. Why did the participant exhibit higher agency in Conversation 2? What factors contributed to the lower coherence and complexity in Conversation 2? If you discussed this and I missed it, my apologies.

**Final note:**

 I hope this feedback is helpful. I typically do not review manuscripts until I fully understand both the spirit and letter of the manuscript, but I am providing this feedback in response to the editor’s request for a timely review

Is the case presented with sufficient detail to be useful for teaching or other practitioners?

Partly

Is the work clearly and accurately presented and does it cite the current literature?

Partly

If applicable, is the statistical analysis and its interpretation appropriate?

Partly

Are all the source data underlying the results available to ensure full reproducibility?

Yes

Are the conclusions drawn adequately supported by the results?

Yes

Is the background of the case’s history and progression described in sufficient detail?

No

Reviewer Expertise:

Youth suicide prevention, qualitative research, technology and social work education/practice

I confirm that I have read this submission and believe that I have an appropriate level of expertise to confirm that it is of an acceptable scientific standard, however I have significant reservations, as outlined above.

# Response

**Published:** 2024-09-07

**Authors:** Chitat CHAN

**Affiliations:** Social Work, Hong Kong Baptist University, Hong Kong, Hong Kong

Responding to Jonathan Singer, Loyola University Chicago, Chicago, Illinois, USA 

 Literature Review

 The literature review begins by emphasizing the potential of AI in narrative interviewing, but it would benefit from first defining and describing what narrative interviewing entails. The enthusiastic discussion of AI, including references to “influential bloggers,” feels more promotional than academic, especially since it turns out to be an advertisement for a for-profit company selling narrative interviewing tools. To strengthen the paper, I recommend moving the definitions of key terms like “narrative interviewing” and “conversation analysis” to the beginning of the literature review. Additionally, information from paragraph 5 and the section “Why do assistant’s interview skills matter” should be relocated to the start to provide important context early on.

 *Response: The literature review part was entirely reorganized. The revised manuscript now includes a more structured literature review. It has been significantly reframed to focus on the democratization of AI chatbots and the emerging trend of customizable AI, which opens new opportunities across various fields, including social work. These advancements enable non-technical users to develop and tailor AI tools for specific applications. As there is limited research on how social workers, especially those with minimal coding experience, can instruct and evaluate AI assistants to conduct narrative interviews, the study investigates this aspect. The role of narrative interviews is instrumental, serving as a case to explore possibilities and provide feedback within a broader context. This sets a clear foundation before discussing the role of AI in narrative interviewing.

 The language in the literature review occasionally seems exaggerated and inconsistent. For instance, the statement that AI is “becoming a crucial tool for researchers” feels overstated without supporting evidence. Furthermore, it appears contradictory to claim that AI improves analysis while also stating that academics have not “explored how AI could enable or limit narrative interviews.” Clarifying these points and providing supporting evidence will strengthen the argument.

 *Response: Improved clarity and exaggeration removed. The revised manuscript moderates such language and focuses on informed claims. It highlights the exploratory nature of the study and emphasizes the use of rubrics as tools for qualitative assessment rather than making broad claims about AI’s capabilities.

 There are also some statements that seem unnecessary and detract from the narrative flow, such as “This brief review of narrative inquirer capabilities is not comprehensive.” Removing such remarks can streamline the text and improve readability.

 *Response: In the revised manuscript, we eliminated remarks such as “This brief review of narrative inquirer capabilities is not comprehensive” to streamline the text and enhance the overall clarity.

 Methods Section

 Introducing a “Methods” header before discussing competence metrics would improve the manuscript’s structure. The justification for the competence metrics needs to be clearer. Rather than stating that “The following metrics were chosen primarily for their simplicity and to facilitate testing and demonstration,” it would be helpful to explain the rationale behind selecting these specific metrics and whether they are based on existing frameworks or newly developed by the authors.

 Scores: The authors identified six scores but did not talk about how those scores were established, the scales, or anything else about them. Specifically:

 Competence 1: The explanation of how progression scores are assigned is vague. There is no clear description of the criteria used to determine the progression stage of each utterance. This lack of specificity makes it difficult to understand the validity and reliability of the scoring system.

 The method’s reliance on previous studies (Au-yeung, 2023; Chan et al., 2020; Connor et al., 2009; Moran et al., 2005; Ramey et al., 2009) without providing a detailed explanation or adaptation process is problematic. It would be helpful to have a more thorough, but not necessarily long explanation of how these methodologies are applied to the current study.

 Relatedly, the sequential analysis method from therapy research may not be directly applicable to narrative interviews conducted by AI. If this method was developed from narrative interviews, then say so. There are therapy approaches like CBT or behavioral therapies that have structured, goal-oriented sessions. ABFT is task oriented, but still provides the therapist structure. Narrative interviews, from your description, are more open-ended, possibly more like Rogerian or supportive therapy sessions. The adaptation of this method needs more rigorous justification.

 I was also unclear how and why a higher score isn’t better, and also why you calculated average progression scores over time. How does this score help us to understand how good AI is doing?

 Competence 2: Is there empirical validation or psychometric testing of the empathy scale? If so, please provide that information for the reader. I would like more information about why a Likert scale is used for empathy, especially since the previous competence argued that higher scores were not necessarily better. That does not seem to be the case with this scale.

 Competence 3: I didn’t understand the use of the word “Domination” in the subheader.

 You introduce multiple metrics (agency, coherence, complexity, and meaning-making) without clearly explaining how they are operationalized. Each one of these needs to be defined and the reader needs measurement criteria for each metric, especially since the previous two measures apparently used different scales. For example, the authors say that measuring “agency” cannot be done in isolation, but there is no description of how the scores or definitions of the other measures are used to understand or measure agency. Indeed, it seems that if the concepts in Competence 3 are interrelated, might there be a way to discuss the measures as a coherent package, rather than individual scales?

 The description of the Case study has many strengths, including the innovative use of ChatGPT to create a chatbot that the researchers would otherwise have not been able to program, highlighting the value of deploying the chatbot on WhatsApp, clearly defining the seven stages, and the ingenuity of having the postgraduate have two conversations – one genuine and one combative. The major limitation of the description of the case study is that it seems to be disconnected from the measures. I know that you provide results in the results section, but it would be helpful to have notes about how each section connects to the measures.

 *Response: We have reframed the entire methodology. The manuscript now features a "Methods" section that explains the autoethnographic approach and the use of rubrics for assessing fidelity and empathy in the AI assistant’s performance. These rubrics provide a qualitative assessment framework that captures the nuances of narrative interviews, reflecting a shift from objective measurement to qualitative assessment aimed at enriching the exploration and discussion. The revised manuscript provides a detailed explanation of how rubrics are used primarily for assessing fidelity and empathy and introduces sequential analysis (and progression scores) to visualize how the conversations aligned with intended plans. The rubrics are the key assessment tools, while the sequential analysis serves more as an illustration. This revised approach emphasizes understanding the context and content of interactions rather than reducing them to numerical scores.

 Data Analysis Method and Data Collection Section

 I was unclear about the context in which the postgrad was having the WhatsApp conversations. Was this in a lab? Were the researchers observing? Was this in private?

 I didn’t understand why the conversation was divided into four equal quarters. i.e. The choice of four quarters seems arbitrary. I know that it makes it easy to analyze by utterances rather than time, but it seems to miss the importance of space and silence in a narrative interview.

 The process for manually assigning progression scores is not clearly defined. There is no description of the criteria used by the human research assistant to assign these scores, which raises concerns about the subjectivity and consistency of the ratings. Is there any research on using LLMs or ChatGPT specifically to assigning scores to narrative interviews? If so, it would be good to cite that here.

 *Response: The WhatsApp conversation took place in private. We have reframed the entire methodology. The manuscript now features a "Methods" section that explains the autoethnographic approach and the use of rubrics for assessing fidelity and empathy in the AI assistant’s performance. The progression scores were primarily instrumental in the visualizations. We used a proportional timeline approach, dividing the conversation into four equal quarters based on the number of utterances instead of actual time, which allows us to account for varying lengths of conversations. For manually assigning progression, the first author and third authors tagged conversations independently, then reviewed and discussed until all disagreements were resolved, which helped ensure reliable and accurate evaluation. The rubrics developed for this study are specifically tailored for narrative interviews and are more flexible than structured, goal-oriented therapy methods. The therapy orientation was de-emphasized. Narrative therapy references were cited because the questioning skills in narrative therapy are useful and help inspire narrative interviews (e.g., creating metaphors). This adaptation is detailed in the methodology section, aligning with the open-ended nature of AI-mediated narrative interviews.

 Results

 The agreement between the human research assistant and ChatGPT4 on most metrics in and of itself is encouraging for researchers interested in using LLMs.

 In finding 2, what distinguishes a 3 from a 4? This is part of my ongoing concern about the paper’s lack of clarity with the measures.

 The disagreement on coherence in Conversation 2 highlights the need for clear criteria. The authors wrote, “In the rating, the human research assistant and ChatGPT4 mostly agreed on all these metrics, except that there was a disagreement on coherence in Conversation 2, where the human research assistant gave a score of 2 and ChatGPT4 gave a score of 3. After reviewing this, the principal researcher sided with ChatGPT4's score.” The manuscript should detail the specific aspects that led to different ratings and the rationale for siding with ChatGPT4’s score. Especially in these early days of using LLMs for analysis, this kind of transparency will be instructive as well as increase trustworthiness of the analysis.

 *Response:  We have completely restructured the methodology. The manuscript now includes a Methods section that explains the autoethnographic approach and the use of rubrics to assess fidelity and empathy in the AI assistant’s performance. We omitted the section on AI tagging, as it no longer aligns with the revised methodology. The rationale for conducting two conversations, as explained in the updated manuscript, was to better assess the extent to which the chatbot maintains similar levels of adherence and fidelity across different contexts, even when the conversation veers off track.  

 Discussion

 I thought it was interesting that there were challenges with long speeches in conversation analysis. Even though WhatsApp tends to have shorter exchanges, what are some potential solutions or alternative methods to address these challenges, especially since narrative interviews often involve extended dialogue. I’m thinking specifically about qualitative researchers who are interested in using this for more traditional open-ended narrative interviews, not on WhatsApp. Perhaps this is part of an expanded discussion on the scalability of this type of AI use.

 I agree that there need to be more refined metrics (and some of my critiques of this paper are in line with that call).

 How would you propose to improve interrater reliability? Again, are there computational linguists who have looked into human/LLM IRR?

 After reading through the discussion a few times, I still didn’t have a good understanding of the differences between conversations 1 and 2. For example, in the results section you noted differences in agency, coherence and complexity. Why did the participant exhibit higher agency in Conversation 2? What factors contributed to the lower coherence and complexity in Conversation 2? If you discussed this and I missed it, my apologies.

 *Response: We have thoroughly restructured the methodology. The revised manuscript adopts an autoethnographic approach and uses rubrics to assess fidelity and empathy in the AI assistant’s performance. These rubrics provide a qualitative framework that captures the nuances of narrative interviews, shifting the focus from objective measurement to a qualitative assessment aimed at deepening exploration and discussion. As such, interrater reliability is no longer a focus in the revised version. The manuscript explains how rubrics are used to assess fidelity and empathy, while sequential analysis and progression scores visually represent how conversations aligned with the intended plans. The rubrics serve as the primary assessment tools, with sequential analysis as an illustration. This approach emphasizes understanding the context and content of interactions rather than reducing them to numerical scores. The revised methodology aims to explore whether an OpenAI Assistant can be effectively instructed by social workers with minimal coding experience, if the chatbot’s performance can be assessed using simple observation-based rubrics, and how these findings contribute to the broader discussion on democratizing AI in specialized fields like social work.

 Final note:

 I hope this feedback is helpful. I typically do not review manuscripts until I fully understand both the spirit and letter of the manuscript, but I am providing this feedback in response to the editor’s request for a timely review

 *Response: We are very thankful for the insightful comments and appreciate the feedback provided. We also want to sincerely apologize, as we were aware that the first version of the manuscript was not fully mature and had several gaps. We recognize that it lacked clarity in several areas, and we are grateful for the opportunity to address those issues. In response, we have completely rewritten the article using the same dataset, but with a more refined focus and clearer articulation of the research aims. This study explores how AI chatbots, particularly those powered by GPT-4, can be applied in social work for conducting narrative interviews. Through an autoethnographic case study approach, social work researchers with limited technical expertise developed and tested an AI chatbot on WhatsApp. The chatbot adhered to a structured interview plan while also demonstrating empathetic engagement with participants. While the AI performed well in maintaining dialogue and showing empathy, challenges were identified in replicating human skills within an AI framework. The study concludes that AI tools can indeed be tailored by social workers to support narrative interviews, though further research is essential to fully address the nuanced needs of the field. Our key focus remains on democratizing AI and empowering domain experts, like social workers, to use these tools effectively. This research enriches the ongoing discussion by exploring the feasibility of AI in specialized fields and emphasizes the importance of interdisciplinary collaboration in developing robust assessment tools to enhance AI implementation.
